# Metabolic Bottlenecks and Opportunities: Reshaping the Tumor Microenvironment for Cancer Immunotherapy

**DOI:** 10.3390/cells15151422

**Published:** 2026-08-05

**Authors:** Jianing Zhang, Zimei Tang, Yiran Wang, Jiaying Wan, Yajing Zhou, Jiexiao Li, Jie Ming

**Affiliations:** 1 Department of Breast and Thyroid Surgery, Union Hospital, Huazhong University of Science and Technology, Wuhan 430022, China; m202376180@hust.edu.cn (J.Z.);; 2 Department of Biochemistry and Molecular Biology, Tongji Medical College, Huazhong University of Science and Technology, Wuhan 430030, China

**Keywords:** tumor microenvironment, metabolic reprogramming, T cell exhaustion, oncometabolites, immunotherapy, epigenetic remodeling

## Abstract

Metabolic reprogramming constitutes a fundamental hallmark of malignancy, orchestrating a hostile tumor microenvironment (TME) that severely compromises anti-tumor immunity. Despite the transformative success of immune checkpoint blockade and adoptive cell therapies, clinical efficacy is frequently curtailed by the metabolic barriers imposed by the TME. This review systematically elucidates the complex metabolic interplay between tumor cells and infiltrating T cells, highlighting two defining mechanisms driving immune evasion: the competitive sequestration of essential nutrients and the accumulation of immunosuppressive oncometabolites. We detail how the depletion of glucose and critical amino acids (glutamine, arginine, methionine, etc.) imposes a state of “metabolic siege” on T cells, impairing their bioenergetics and effector functions. Concurrently, we explore how accumulated metabolites—such as lactate, succinate, 2-hydroxyglutarate, kynurenine, and lipids—function as non-canonical signaling molecules to subvert immune surveillance via epigenetic remodeling and oxidative stress. Furthermore, we synthesize emerging therapeutic strategies designed to dismantle this metabolic barrier, including targeting metabolic enzymes (IDO1 and FASN) and transporters, repurposing metabolic waste, and genetically engineering T cells with enhanced metabolic fitness and resilience. By integrating the latest insights into the “metabolism–epigenetics–immunity” axis, this review provides a theoretical foundation for developing next-generation immunotherapies that target metabolic vulnerabilities to overcome resistance in cancer treatment.

## 1. Introduction

Metabolic reprogramming is a recognized hallmark of cancer that extends beyond intrinsic shifts in tumor cell metabolism; it encompasses the metabolic remodeling of stromal cells and the unique metabolic microenvironment shaped by these components collectively [1]. Recently, we have come to appreciate the complex interplay among nutrients, metabolites, and immune cells within this niche, recognizing their pivotal roles in driving immune suppression and tumor evasion [2,3]. Driven by the Warburg effect and other oncogenic metabolic programs, tumor cells extensively reprogram glucose, amino acid, and lipid metabolism to sustain rapid proliferation, biosynthetic demands, and redox homeostasis [4]. Moreover, core metabolic pathways governing amino acids and lipids are aberrantly reprogrammed to support biosynthetic requirements and maintain redox homeostasis. This metabolic rewiring creates intense competition for essential nutrients while simultaneously driving the accumulation of immunosuppressive metabolites, collectively reshaping the metabolic fitness, phenotype, and effector functions of infiltrating immune cells and ultimately promoting immune evasion and tumor progression [2]. For instance, tumor-derived succinate promotes the M2 polarization of tumor-associated macrophages (TAMs) and cancer cell migration via SUCNR1 signaling [5]. Similarly, lactate impairs the function of effector T lymphocytes (Teff) through various mechanisms, while altering the metabolism of regulatory T lymphocytes (Tregs) to sustain their activity within the glucose-deprived environment [6]. Concurrently, these immune cells do not merely respond to tumor cell metabolic demands; they actively engage in a complex network of metabolic dependencies with tumor and stromal cells through nutrient competition, metabolite secretion, and reciprocal signaling [1,7]. Together, these interactions support viewing the TME as an adaptive metabolic ecosystem rather than a collection of isolated pathways. The extensive metabolic crosstalk among tumor cells, stromal cells, and immune cells not only drives immune evasion but also represents a major obstacle to the successful clinical translation of metabolism-targeted therapies [2].

Understanding the metabolic landscape of the TME is essential not only for elucidating mechanisms of tumor progression but also for overcoming one of the major barriers to effective cancer immunotherapy [2]. Although immune checkpoint blockade (ICB) and adoptive cell therapy (ACT) have revolutionized cancer treatment, their long-term efficacy still relies on the ability of effector T cells to infiltrate, persist, and maintain metabolic fitness within the TME [7,8]. However, nutrient deprivation, hypoxia, extracellular acidosis, and the accumulation of immunosuppressive metabolites collectively drive T cell dysfunction, ultimately limiting durable therapeutic responses. Importantly, despite substantial progress in identifying metabolic vulnerabilities within the TME, the clinical translation of metabolism-targeted therapies has been considerably slower than anticipated. This limited success largely reflects the remarkable metabolic plasticity of both tumor and immune cells, as well as the extensive crosstalk and compensatory interactions among multiple metabolic pathways. Therefore, rather than considering individual metabolic pathways in isolation, a comprehensive understanding of their coordinated regulation is essential for developing more effective metabolic interventions. In this review, we integrate recent advances in glucose, amino acid, and lipid metabolism, discuss emerging evidence on metabolic crosstalk and translational challenges, and summarize current approaches to reshaping the metabolic microenvironment and improving anti-tumor immunity.

## 2. Nutrient Metabolism in the Tumor Microenvironment

### 2.1. Glucose Metabolites in the Tumor Microenvironment

#### 2.1.1. Glucose: A Battleground of Multilateral Competition

Even under aerobic conditions, tumor cells preferentially utilize aerobic glycolysis (the Warburg effect) to support rapid proliferation and anabolic biosynthesis rather than maximizing ATP production [9]. This metabolic adaptation not only fulfills the energetic and biosynthetic demands of malignant cells but also fundamentally reshapes nutrient availability within the TME, creating a competitive metabolic environment that places infiltrating immune cells at a distinct disadvantage [2]. Tumor cells coordinately upregulate GLUTs and key glycolytic enzymes to sustain high glycolytic flux, thereby securing a competitive advantage in glucose acquisition within the TME [10]. Conventionally, glucose deprivation within the tumor microenvironment (TME) was attributed to excessive consumption by tumor cells. However, recent studies have challenged this view, revealing that tumor-associated myeloid cells—including tumor-associated macrophages (TAMs) and monocytic myeloid-derived suppressor cells (M-MDSCs)—exhibit the highest capacity for glucose uptake. In contrast, tumor cells display a distinct preference for the uptake of glutamine and lipids [11]. These findings suggest that glucose competition within the TME is not exclusively dictated by tumor cells but instead reflects a multicellular metabolic network involving both malignant and immune populations. Consistent with these findings, the development and function of myeloid cells within the TME are indeed strictly dependent on glycolysis [12]. However, T cells also upregulate glycolysis during activation to meet the bioenergetic and biosynthetic demands required for rapid clonal expansion [13]. Consequently, glucose availability within the TME is determined by dynamic metabolic competition among tumor cells, myeloid cells, and effector T cells rather than by tumor cell consumption alone. Quantitative analysis reveals that, within the tumor mass, approximately two-thirds of glucose is consumed by tumor cells and one-third by myeloid cells, leaving negligible amounts for other immune cell populations [11]. This glucose deprivation forces a shift in the T cell metabolic profile, impairing mTOR activity and IFN-γ production, which ultimately facilitates tumor progression. Notably, checkpoint blockade antibodies targeting CTLA-4, PD-1, and PD-L1 have been shown to dampen tumor cell glycolysis and restore glucose levels in the TME, thereby reinvigorating T cell glucose uptake and IFN-γ production [14]. These findings suggest that metabolic reprogramming may represent an important mechanism underlying the therapeutic efficacy of immune checkpoint blockade. More importantly, they support the rationale for combining metabolic interventions with immunotherapy, although the optimal targets and treatment strategies remain to be established.

#### 2.1.2. Lactate: A Metabolic Boon for Pro-Tumor Cells but a Bane for Anti-Tumor Immunity

Under hypoxic conditions, glucose is converted into pyruvate via glycolysis, which is subsequently reduced to lactate by lactate dehydrogenase (LDH). Driven by the Warburg effect, tumor cells rapidly metabolize glucose into copious amounts of lactate even in the presence of ample oxygen. Through monocarboxylate transporters (MCTs), tumor cells export excess cytosolic lactate into the extracellular space, precipitating lactate accumulation and a reduction in pH within the microenvironment [6]. Although traditionally regarded as a metabolic waste product, lactate is now recognized as an important metabolic fuel that supports mitochondrial metabolism and redox homeostasis in tumor cells [15,16,17]. These findings have fundamentally changed the perception of lactate from a metabolic by-product to a multifunctional metabolite that actively promotes tumor adaptation under metabolic stress. Beyond its role as a fuel, lactate acts as a signaling molecule that activates the lactate receptor (GPR81) on tumor cells, orchestrating a cascade of biological effects to support tumor growth [18]. In breast cancer, GPR81 activation triggers the PI3K/AKT/CREB signaling axis, promoting proliferation and angiogenesis [19]. Concurrently, GPR81 upregulates the expression of BRCA1 and ABCB1, conferring resistance to chemotherapy [20]. These observations establish lactate as both a metabolic substrate and a signaling hub that coordinates multiple tumor-promoting pathways. Non-malignant pro-tumor cells also thrive on lactate. Regulatory T cells (Tregs) utilize MCT1 to harvest lactate from the TME, channeling it into the TCA cycle [21]. Intracellular lactate further modulates RNA splicing to sustain the phenotype and function of tumor-infiltrating Tregs [22]. Additionally, macrophage uptake of extracellular lactate leads to histone lactylation—a modification on lysine residues—which upregulates Arg1 expression and drives differentiation toward an M2-like phenotype [23]. Similarly, lactate within the TME activates GPR132 on macrophages, skewing them toward M2 polarization [24]. Moreover, studies indicate that the lactate-rich milieu fosters the recruitment of myeloid-derived suppressor cells (MDSCs) and mediates an immunosuppressive microenvironment [25]. These observations demonstrate that lactate selectively supports immunosuppressive cell populations while simultaneously impairing anti-tumor immunity, thereby amplifying immune tolerance within the TME. This dual role highlights lactate as one of the central metabolic regulators of tumor immune escape.

In stark contrast, lactate exerts deleterious effects on immune cells critical for anti-tumor immunity. Upon entry into CD8^+^ T cells, lactate downregulates pyruvate carboxylase expression, depleting TCA cycle intermediates and ultimately suppressing CD8^+^ T cell function [26]. Furthermore, tumor-derived lactate inhibits LDH, thereby compromising autophagy and precipitating excessive reactive oxygen species (ROS) generation in naive T cells, which further impairs their survival and function [27]. Lactate also abrogates signaling pathways involving the nuclear factor of activated T cells (NFAT) in T cells and natural killer (NK) cells, diminishing IFN-γ production and threatening cell viability [28]. In NK cells, lactate influx via MCTs lowers intracellular pH, dampening glycolytic activity and cytotoxic function, and potentially triggering apoptosis [29]. Additionally, tumor-derived lactate interferes with dendritic cell (DC) activation and antigen presentation by suppressing Type I interferon signaling downstream of TLR3 and STING, thereby accelerating antigen degradation [30,31]. Importantly, the suppressive effects of lactate extend across both innate and adaptive immunity, suggesting that lactate accumulation reshapes the immune landscape at multiple levels rather than affecting individual immune cell subsets in isolation.

Overall, lactate accumulation compromises effector T-cell, NK-cell, and dendritic-cell function while supporting tumor cells and immunosuppressive populations, thereby establishing a metabolically privileged niche for tumor progression. Given its central role in coordinating both tumor metabolism and immune suppression, targeting lactate metabolism has emerged as an attractive therapeutic strategy. Nevertheless, because lactate also participates in normal immune cell metabolism, selectively disrupting tumor-associated lactate signaling without impairing anti-tumor immunity remains a major challenge for clinical translation.

#### 2.1.3. Succinate: Distinct Roles Across Intracellular and Extracellular Compartments

Succinate serves as a pivotal intermediate in the tricarboxylic acid (TCA) cycle, generated from succinyl-CoA via succinyl-CoA synthetase. Concurrently, it functions as the substrate for succinate dehydrogenase (SDH), which catalyzes its oxidation to fumarate while transferring electrons to Complex II of the electron transport chain (ETC) [32]. SDH is a four-subunit enzyme complex responsible for oxidizing succinate to fumarate. Genetic mutations or functional loss of SDH have been reported in multiple malignancies, resulting in succinate accumulation, impaired oxidative phosphorylation, enhanced tumor invasiveness, and poor clinical prognosis [33,34,35,36,37]. Mechanistically, intracellular succinate acts as a competitive inhibitor of prolyl hydroxylases (PHDs) by structurally mimicking their co-substrate, α-ketoglutarate. Since PHDs are responsible for hydroxylating proline residues on HIF-1α to mark it for proteasomal degradation, succinate accumulation stabilizes HIF-1α, thereby inducing the transcription of genes governing glycolysis and angiogenesis that ultimately fuel cancer progression [38]. However, this pathway is also critical for maintaining the pro-inflammatory phenotype of macrophages. In classically activated macrophages (M1), SDH is inhibited by itaconate, leading to succinate accumulation, which subsequently stabilizes HIF-1α and drives IL-1β expression [39]. These observations illustrate a context-dependent role of intracellular succinate. While succinate accumulation promotes tumor progression through HIF-1α stabilization in cancer cells, it is also required for sustaining inflammatory activation in M1 macrophages, suggesting that therapeutic targeting of succinate metabolism may produce distinct effects across different cell populations.

Beyond its intracellular roles, succinate can be exported from the mitochondrial matrix to the cytosol via the mitochondrial dicarboxylate carrier (SLC25A10) [40], and subsequently released into the extracellular space through plasma membrane transporters of the SLC13 family via mechanisms that remain incompletely understood [41]. Elevated succinate levels have been detected in the culture supernatants of lung, breast, prostate, and colon cancer cell lines, as well as in tumor tissues and biofluids from patients with various malignancies [5,42]. Within the tumor microenvironment (TME), extracellular succinate functions as a signaling molecule (an “oncometabolite”) by binding to the succinate receptor 1 (SUCNR1, also known as GPR91), activating diverse signaling cascades [5,43,44]. The effects of extracellular succinate vary across cellular compartments, partly because of the diversity of SUCNR1 downstream signaling. In tumor cells, SUCNR1 activation promotes migration via the PI3K-Akt pathway [5], whereas in macrophages, extracellular succinate predominantly drives M2 polarization through SUCNR1-mediated Gq signaling [5,44]. Additionally, gastric cancer-derived succinate induces endothelial cell proliferation and upregulates VEGF expression via the STAT3-ERK1/2 axis, fostering tumor angiogenesis [45]. Aside from receptor-mediated signaling, succinate in the TME can directly enter cells to exert physiological effects. One study demonstrated that extracellular succinate is taken up by T cells via MCT1, where it inhibits succinyl-CoA synthetase and suppresses TCA cycle flux, thereby impairing the function of CD4^+^ and CD8^+^ T cells [46]. Conversely, another study reported that SUCNR1 signaling enhances cytotoxic molecule production and promotes anti-tumor activity in T cells [47]. These apparently contradictory findings demonstrate that succinate functions as both a metabolic intermediate and an immunoregulatory signal, with its effects determined by cellular origin, subcellular localization, receptor engagement, and immune-cell metabolic state. Resolving these variables will be essential for the rational development of succinate-targeted therapies and may explain why such interventions have not yet achieved consistent translational success.

#### 2.1.4. Fumarate: Widespread Immunosuppressive Effects

Fumarate (also known as fumaric acid) is another critical intermediate of the TCA cycle, primarily generated via the oxidative dehydrogenation of succinate catalyzed by SDH. Besides the TCA cycle, fumarate also participates in several metabolic pathways and is normally converted to malate by fumarate hydratase (FH) [48]. Defects or mutations in FH are closely linked to the oncogenesis of certain tumors [49]. Studies indicate that intracellular fumarate accumulation resulting from FH deficiency inhibits α-KG-dependent DNA and histone demethylases, leading to epigenetic hypermethylation, activation of EMT-associated transcriptional programs, and enhanced invasiveness of renal cancer cells [50]. Furthermore, fumarate can induce the succination of cysteine residues within Kelch-like ECH-associated protein 1 (KEAP1), abrogating its ability to inhibit the Nuclear factor erythroid 2-related factor 2 (NRF2)-mediated antioxidant response pathway, thus promoting tumorigenesis [51]. Importantly, fumarate-mediated succination is mechanistically distinct from succinate-mediated succinylation. Whereas succination is an irreversible covalent modification of cysteine residues, succinylation is a reversible lysine acylation process [52,53]. Fumarate therefore promotes tumor progression through epigenetic remodeling and redox adaptation, extending its role beyond that of a conventional TCA-cycle intermediate.

Historically, however, the investigation of fumarate did not originate in oncology but rather in the study of membrane-permeable fumarate esters. Membrane-permeable fumarate esters, such as dimethyl fumarate (DMF), have long been used to treat autoimmune diseases because of their potent immunomodulatory activity [54,55]. After entering cells, DMF is converted into fumarate, which suppresses glycolysis by inducing GAPDH succination, thereby limiting macrophage and T cell activation [56]. Moreover, fumarate can directly bind to and inhibit death-associated protein kinase 1 (DAPK1), thereby impairing the anti-tumor function of CD8^+^ T cells [57]. Interestingly, these findings appear paradoxical when compared with the well-established anti-inflammatory effects of dimethyl fumarate (DMF) in autoimmune diseases. This discrepancy likely reflects the dependence of fumarate activity on its source, concentration, intracellular localization, and responding cell type. Consequently, therapeutic strategies based on fumarate modulation should carefully distinguish between pharmacological administration of fumarate esters and pathological fumarate accumulation within the TME.

Recent insights have further unveiled the regulatory role of extracellular fumarate on immune cell function. Fumarate accumulated within tumor cells can be released into the extracellular space, elevating fumarate levels within the tumor microenvironment (TME) [48]. Despite being a polar molecule, tumor-derived fumarate can infiltrate T cells and induce succination at the C96 and C102 residues of ZAP70. This modification impairs T cell receptor (TCR) signal transduction, thereby inhibiting CD8^+^ T cell activation and anti-tumor immune responses [58]. These findings extend the immunosuppressive role of fumarate beyond intracellular metabolic regulation, suggesting that extracellular fumarate may function as an intercellular metabolic signal capable of directly reshaping anti-tumor immunity. Although specific fumarate receptors and transporters remain unidentified, it is postulated that fumarate may enter cells via certain carboxylate transporters. Current evidence therefore supports dual roles for fumarate as an intracellular metabolic regulator and an extracellular immunomodulatory metabolite. However, many fundamental questions remain unresolved, including the mechanisms governing extracellular fumarate transport, its potential receptor-mediated signaling, and how these processes may be therapeutically targeted without disrupting physiological metabolism. Addressing these knowledge gaps will be essential for translating fumarate-targeted strategies into clinical practice.

#### 2.1.5. 2-Hydroxyglutarate: Distinct Immune Effects of the Two Enantiomers

2-Hydroxyglutarate (2HG) exists as two distinct enantiomers: D-2HG and L-2HG (also designated as R-2HG and S-2HG, respectively). D-2HG is primarily generated by neomorphic IDH1/2 mutations, whereas L-2HG is mainly produced under hypoxic or acidic conditions through alternative metabolic reactions involving LDHA [59]. Owing to their structural similarity to α-KG, both D-2HG and L-2HG inhibit α-KG-dependent dioxygenases, resulting in widespread epigenetic remodeling that contributes to tumorigenesis and establishes 2HG as a representative oncometabolite [48].

Crucially, however, these two enantiomers exert diametrically opposed effects on T cell function within the tumor microenvironment. D-2HG significantly abrogates the migration, proliferation, and cytokine secretion of activated T cells [60]. Mechanistically, tumor-derived D-2HG enters CD8^+^ T cells through SLC13A3 and suppresses T cell activation by disrupting NFAT signaling, impairing mitochondrial metabolism, and inhibiting glycolytic activity [61,62]. Beyond functional suppression, D-2HG skews T cell differentiation fates. Elevated levels of D-2HG induce hypermethylation at the Foxp3 locus—the master transcription factor for regulatory T cells (Tregs)—thereby repressing Treg differentiation while reciprocally promoting Th17 lineage commitment. Conversely, reducing D-2HG levels via aminooxyacetic acid treatment restores Foxp3 expression and fosters Treg generation [63]. Consistent with this, clinical observations in IDH-mutant gliomas reveal a reduced proportion of Tregs among infiltrating T cells [64]. In stark contrast, intracellular L-2HG levels are naturally elevated in activated CD8^+^ T cells in vitro [65]. Strikingly, Chimeric Antigen Receptor T (CAR-T) cells treated with L-2HG exhibit enhanced proliferative capacity, superior persistence, and potent anti-tumor efficacy in vivo, a phenomenon validated in tumor-bearing mouse models [66]. These beneficial effects are likely attributed to L-2HG-mediated modulation of histone H3K4 and DNA methylation, as well as the stabilization of HIF-1α [65]. The divergence in cellular outcomes between D-2HG and L-2HG may stem from their differential inhibitory potencies against specific α-KG-dependent enzymes: L-2HG is a more potent inhibitor of HIF prolyl hydroxylases (leading to HIF-1α stabilization), whereas D-2HG is a more effective inhibitor of histone demethylases [67]. More importantly, these findings suggest that 2HG should not be regarded as a single metabolic entity. Instead, its biological functions are highly dependent on stereochemistry, cellular origin, and metabolic context, highlighting the importance of distinguishing between D-2HG and L-2HG in both mechanistic studies and therapeutic development.

Regarding myeloid cells, while D-2HG does not appear to affect dendritic cell differentiation or antigen presentation [60], tumor-derived D-2HG enhances the conversion of tryptophan to kynurenine in macrophages. This metabolite accumulation activates the aryl hydrocarbon receptor (AhR), driving macrophage differentiation toward an immunosuppressive phenotype [68]. Given the disparate impacts of D-2HG and L-2HG on T cell function and differentiation, future evaluations of immune status in patients with IDH1/2-mutant cancers must rigorously distinguish between these isomers to delineate their specific functional contributions.

#### 2.1.6. Itaconate: An Emerging Alkylating and Acylating Immuno-Metabolite in the Myeloid-Rich TME

Itaconate acts as a shunt metabolite of the tricarboxylic acid (TCA) cycle, generated via the decarboxylation of cis-aconitate catalyzed by aconitate decarboxylase 1 (ACOD1), which is encoded by the immune response gene 1 (IRG1) [69]. Early investigations into itaconate focused predominantly on macrophage metabolism. Itaconate production and secretion are markedly upregulated in classically activated macrophages (M1), playing a pivotal role in the metabolic reprogramming associated with polarization. Due to its structural homology with succinate, itaconate acts as a competitive inhibitor for the SDH binding site. This inhibition precipitates succinate accumulation and constitutes the mechanistic basis for the “SDH break” observed in the TCA cycle of M1 macrophages [70]. In addition, itaconate modifies cysteine residues on KEAP1, leading to NRF2 activation and the induction of antioxidant and anti-inflammatory gene programs [69,71]. This effect strikingly mirrors the KEAP1 succination mediated by fumarate. More recently, however, it was discovered that itaconate can also induce an acylation reaction with lysine residues, resulting in a novel post-translational modification known as itaconylation [72]. Itaconate thus acts not only as a metabolic intermediate but also as a molecular link between cellular metabolism and inflammatory signaling. However, many of its downstream regulatory mechanisms remain incompletely understood, particularly the physiological significance of itaconylation, highlighting an important area for future investigation.

In recent years, itaconate has emerged as a significant tumor metabolite, a recognition stemming from the association between IRG1 expression and various malignancies. IRG1 expression correlates with tumor staging in glioma patients, and its overexpression has been shown to foster glioma cell proliferation and invasion [73]. Unlike most oncometabolites, which are predominantly produced by tumor cells, itaconate within the tumor microenvironment (TME) appears to originate mainly from myeloid cells, particularly tumor-associated macrophages (TAMs) and myeloid-derived suppressor cells (MDSCs) [74,75,76]. Notably, Irg1 deficiency in mice leads to an expansion of M1 macrophages within the TME and enhanced antigen-presenting capacity, while simultaneously promoting CD8^+^ T cell infiltration [75]. Furthermore, recent studies reveal that myeloid-derived suppressor cells (MDSCs) secrete copious amounts of itaconate into the TME. Upon uptake by CD8^+^ T cells, this extracellular itaconate perturbs the biosynthesis of aspartate, serine, and glycine, thereby suppressing T cell proliferation and effector function [77]. These observations further position itaconate as a mediator of the interaction between myeloid-cell metabolism and adaptive immune suppression, highlighting myeloid metabolic reprogramming as a potential therapeutic target.

**Figure 1:**
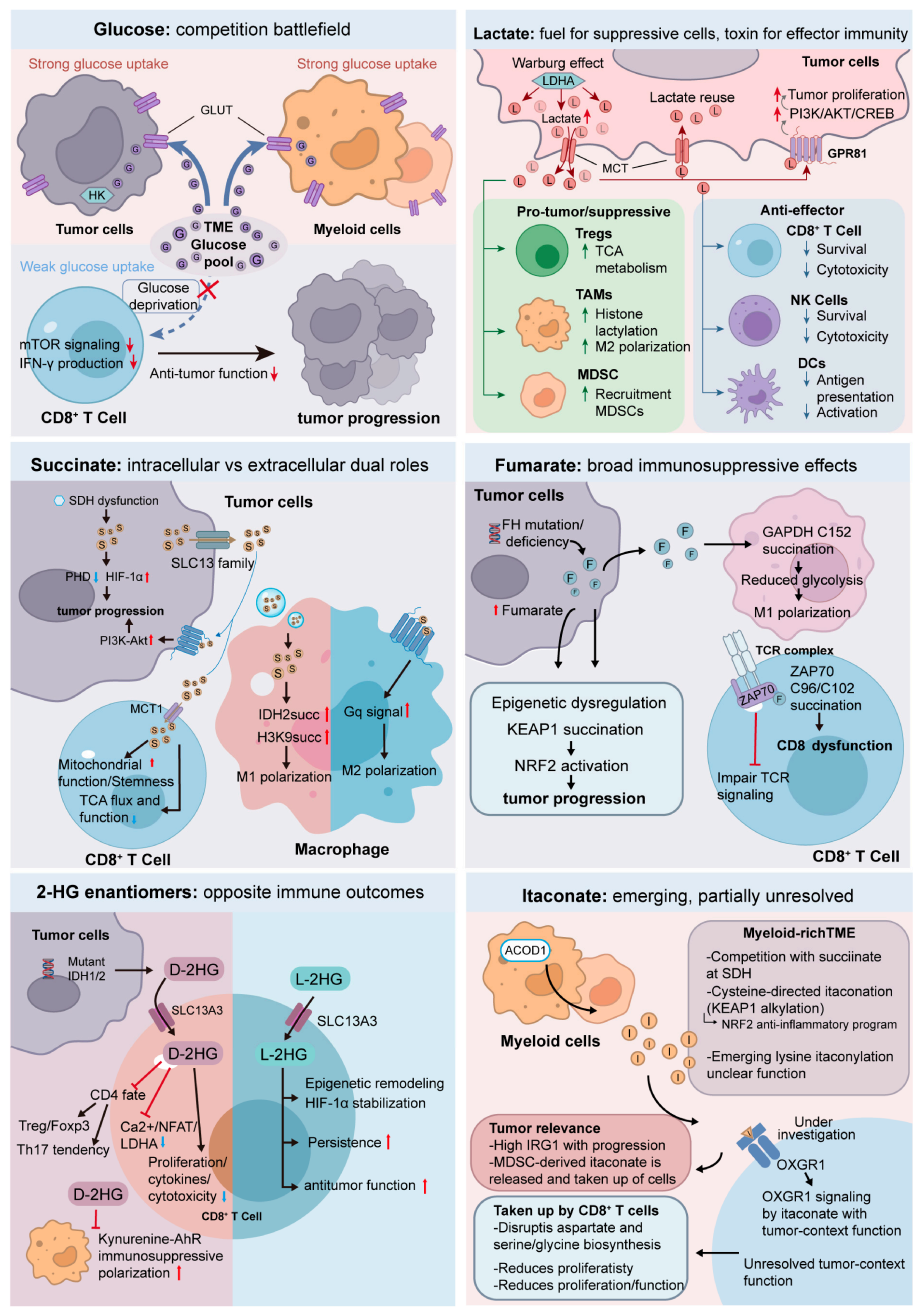
**Metabolic compartmentalization and crosstalk within the tumor microenvironment (TME).** Tumor-driven metabolic reprogramming and TCA cycle aberrations generate a nutrient-depleted, waste-enriched niche that profoundly orchestrates immune cell fate and function. **Glucose:** Tumor and myeloid cells aggressively sequester glucose via upregulated GLUTs, imposing a “metabolic siege” on CD8^+^ T cells that blunts mTOR signaling and IFN-γ production, thereby impairing anti-tumor immunity. **Lactate:** Aerobic glycolysis generates abundant lactate, which serves as an alternative TCA fuel and epigenetic modifier (e.g., histone lactylation) for immunosuppressive lineages including Tregs, TAMs, and MDSCs. Conversely, it acts as a potent toxin that abrogates the survival, cytotoxicity, and antigen presentation of effector T cells, NK cells, and DCs. **Succinate:** Intracellularly, SDH dysfunction triggers succinate accumulation, stabilizing HIF-1α to drive tumor progression. Extracellularly, secreted succinate engages the SUCNR1 (GPR91) receptor to promote M2 macrophage polarization (via Gq signaling) and tumor proliferation (via the PI3K-Akt axis). **Fumarate:** FH deficiency causes fumarate buildup, leading to widespread epigenetic dysregulation and the covalent succination of critical targets. This includes KEAP1 alkylation in tumor cells to activate NRF2, and ZAP70 succination (at C96/C102) in CD8^+^ T cells, which profoundly blunts TCR signal transduction. **2-HG Enantiomers:** Tumor-derived D-2HG suppresses Treg differentiation while promoting Th17 lineage commitment and impairing CD8^+^ T cell activation. In stark contrast, T cell-intrinsic L-2HG promotes beneficial epigenetic remodeling and HIF-1α stabilization, thereby enhancing CD8^+^ T cell persistence and anti-tumor efficacy. **Itaconate:** In the myeloid-rich TME, ACOD1-mediated itaconate synthesis not only exerts anti-inflammatory programs via KEAP1 alkylation but also directly suppresses CD8^+^ T cell function by disrupting aspartate and serine/glycine biosynthesis. Its emerging roles via lysine itaconylation and OXGR1 signaling remain under active investigation.

Beyond its intracellular metabolic functions, itaconate has also been proposed to act as an extracellular signaling molecule through oxoglutarate receptor 1 (OXGR1, also known as GPR99). Itaconate-mediated OXGR1 signaling has been implicated in pulmonary innate immune responses and the regulation of airway epithelial function [78]. However, the physiological relevance of this signaling pathway remains controversial. OXGR1 was originally identified as a receptor for α-ketoglutarate and can also be activated by cysteinyl leukotrienes (CysLTs), suggesting that receptor activation may depend on ligand availability and tissue context rather than a single endogenous agonist [79,80]. Consequently, whether OXGR1 serves as a bona fide itaconate receptor within the tumor microenvironment remains unresolved. Future studies should clarify the ligand specificity, downstream signaling, and immunological consequences of the itaconate–OXGR1 axis before this pathway can be considered a viable therapeutic target (Figure 1).

### 2.2. The Amino Acid Wasteland: Depletion and Nutrient-Sensing Subversion

Driven by rapid proliferation and elevated biosynthetic demands, tumor cells frequently upregulate the expression of amino acid transporters to facilitate the high-throughput uptake and consumption of amino acids [81,82]. This aggressive consumption precipitates a scarcity of various amino acids within the tumor microenvironment (TME), compelling all resident cells to compete for limited resources to sustain viability. However, immune cells—particularly T cells—often find themselves ill-equipped to adapt to this fierce competition, succumbing to a state of “metabolic siege”. Ample evidence indicates that amino acid deprivation severely hampers T cell activation, thereby compromising anti-tumor immunity [83]. The mechanistic impact of amino acid starvation on anti-tumor immunity is exerted primarily through the following five pathways: (1) The mammalian target of rapamycin (mTOR) signaling pathway, specifically responding to leucine, arginine, lysine, glutamine, methionine, and tryptophan. (2) The general control nonderepressible 2 (GCN2) signaling pathway, a key sensor for amino acid sufficiency. (3) Immune checkpoint modulation, regulating the expression levels of PD-1/PD-L1. (4) Metabolic–epigenetic coupling, influencing gene expression via histone modifications and DNA methylation. (5) Metabolic network hub functions, dictating the rigid requirements for specific amino acids in fundamental biological processes, such as nucleotide synthesis and the maintenance of redox homeostasis [84].

The subsequent sections will systematically delineate the amino acids that undergo significant metabolic alterations within the TME, focusing on their pivotal regulatory roles in shaping the anti-tumor immune response.

#### 2.2.1. Glutamine: Monopolization by Malignant Cells and Foxp3 Epigenetic Skewing

Glutamine, the most abundant amino acid in human plasma, serves as a major bioenergetic substrate together with glucose and provides nitrogen for nucleotide, lipid, and glutathione synthesis, while replenishing the tricarboxylic acid (TCA) cycle through anaplerosis [85,86,87]. Its cellular entry is facilitated by specific membrane transporters, most notably SLC1A5, SLC38A1, and SLC38A2 [88].

Tumor cells frequently upregulate these transporters to sequester massive amounts of glutamine, satisfying the immense biosynthetic and bioenergetic demands imposed by rapid proliferation [88]. Notably, the overexpression of SLC1A5, SLC38A2, and SLC38A5 has been validated in clinical specimens across a spectrum of malignancies [89]. Although activated T cells also upregulate SLC1A5 to enhance their glutamine uptake capacity [90], quantitative studies reveal that over 90% of intratumoral glutamine is consumed by cancer cells within the tumor microenvironment (TME) [11]. This places tumor-infiltrating T cells (TILs) at a distinct competitive disadvantage, subjecting them to severe glutamine deprivation. Yet, glutamine is indispensable for T cell activation, clonal expansion, and differentiation into effector phenotypes [91,92]. Specifically, the expression of activation markers (CD25, CD45RO, and CD71) and the production of functional cytokines (IFN-γ and TNF-α) are strictly glutamine-dependent [93]. Glutamine deficiency resulting from SLC1A5 ablation has been shown to impair Th1 and Th17 differentiation and suppress inflammatory T cell responses in autoimmune models [90]. Concurrently, glutamine restriction during T cell activation promotes Foxp3 expression and skews differentiation toward regulatory T cells (Tregs) [94]. Mechanistically, glutamine restriction reduces intracellular α-KG availability and alters α-KG-dependent epigenetic remodeling at T cell lineage-associated loci, thereby favoring Foxp3 expression and Treg differentiation while limiting Th1 commitment [95]. However, accumulating evidence suggests that the immunological consequences of glutamine restriction are highly context-dependent. While chronic glutamine deprivation compromises effector T cell function, transient or pharmacological glutamine blockade has been reported to promote the generation of long-lived, memory-like T cells with improved persistence and anti-tumor activity [96]. Beyond T cells, glutamine scarcity within the TME may also impede B cell differentiation into plasma cells and limit lymphoblast conversion [97]. Additionally, glutamine is intimately linked to superoxide generation and the subsequent formation of neutrophil extracellular traps (NETs) in neutrophils [98]. These apparently divergent observations suggest that the immunological effects of glutamine depend less on its absolute availability than on the capacity of individual immune subsets to adapt to glutamine restriction. Defining this metabolic flexibility will be important for developing interventions that preserve anti-tumor immunity while disrupting tumor glutamine dependence.

#### 2.2.2. Arginine: A Double-Edged Sword for Tumor Survival and Immunity

Arginine is a semi-essential amino acid that becomes conditionally indispensable during rapid growth, inflammation, and tumor progression [99]. It participates in multiple metabolic pathways, most notably arginase (ARG)-mediated urea metabolism and nitric oxide synthase (NOS)-dependent nitric oxide production [99]. Increased expression of ARG1 and ARG2 has been reported in multiple malignancies, indicating that enhanced arginine metabolism is a characteristic feature of the tumor microenvironment (TME) [89].

Crucially, arginine plays a pivotal role in T cell survival, proliferation, differentiation, cytokine production, and effector function. Supplementation with exogenous arginine has been shown to enhance the survival and anti-tumor immunity of both CD4^+^ and CD8^+^ T cells [100]. Mechanistically, research indicates that ARG2 produced by murine renal cell carcinoma precipitates arginine depletion, which downregulates the expression of the TCR CD3ζ chain in T cells, thereby impairing their signaling competence [101]. Similarly, MDSC-derived ARG1 induces arginine depletion that activates the GCN2 stress response signaling pathway, arresting T cells in the G0/G1 phase and blocking their entry into the S phase, thus inhibiting proliferation [102]. Paradoxically, many tumor cells are themselves arginine auxotrophs and therefore depend on extracellular arginine to sustain proliferation [103]. This creates a unique therapeutic dilemma: arginine depletion suppresses both tumor growth and anti-tumor immunity, whereas arginine supplementation enhances T cell function but may simultaneously support tumor cell proliferation. Current evidence suggests that the anti-tumor effects of arginine deprivation generally outweigh its immunosuppressive consequences in arginine-auxotrophic tumors. Nevertheless, these findings also indicate that arginine metabolism cannot be manipulated indiscriminately. Accordingly, arginine-targeted interventions may require combination with immunotherapy or cell-selective modulation of arginine metabolism to maximize therapeutic benefit [104].

#### 2.2.3. Serine: At the Crossroads of Biosynthetic Networks and Anti-Tumor Immunity

Serine is obtained from dietary intake or synthesized de novo from the glycolytic intermediate 3-phosphoglycerate (3-PG) or through the reversible conversion of glycine by serine hydroxymethyltransferase (SHMT). As a central metabolic intermediate, serine supports lipid biosynthesis, glutathione production, and one-carbon metabolism, thereby providing essential precursors for nucleotide synthesis, NADPH generation, and S-adenosylmethionine (SAM) production [105].

Dietary serine and glycine restriction profoundly reshapes tumor metabolism. In response to nutrient limitation, tumor cells activate the serine synthesis pathway (SSP), increase oxidative phosphorylation, and accumulate reactive oxygen species (ROS), rendering metabolically stressed tumors—particularly those with p53 deficiency—more susceptible to growth inhibition [106]. Consistently, key SSP enzymes, including PHGDH, PSAT1, and PSPH, are frequently upregulated in human cancers, underscoring the importance of endogenous serine synthesis for tumor progression [107,108,109]. Nevertheless, endogenous synthesis is often insufficient to fully compensate for serine deprivation, and dietary serine/glycine restriction has therefore demonstrated anti-tumor efficacy in several preclinical models [110,111].

However, serine restriction produces markedly different outcomes across immune-cell subsets. Effector CD8^+^ T cells rely heavily on exogenous serine to sustain one-carbon metabolism, mitochondrial biogenesis, nucleotide synthesis, and clonal expansion [112,113]. Accordingly, serine deficiency markedly impairs CD8^+^ T cell proliferation and effector function. In contrast, regulatory T cells (Tregs) appear to better adapt to serine/glycine-restricted conditions, thereby maintaining or even enhancing their suppressive activity, potentially through glutathione-dependent metabolic remodeling [114]. These findings highlight a fundamental therapeutic dilemma: although serine/glycine restriction suppresses tumor growth, it may simultaneously compromise anti-tumor immunity by limiting effector T cell responses while favoring immunosuppressive Treg populations. These observations suggest that metabolic plasticity differs substantially among immune cell subsets. Therefore, therapeutic manipulation of serine metabolism should focus on understanding the metabolic adaptability of individual cell populations rather than simply restricting systemic serine availability.

#### 2.2.4. Tryptophan-Kynurenine: The Subversion of an Essential Nutrient into an Immunosuppressive Signal

Tryptophan (Trp) is an essential amino acid acquired exclusively through dietary intake. Extracellular tryptophan is transported into cells via the neutral amino acid transporter SLC7A5. Once intracellular, a minor fraction is utilized for protein and neurotransmitter (e.g., serotonin) synthesis, while over 95% is shunted into the kynurenine pathway [115,116]. Tumor cells, tumor-associated macrophages (TAMs), and subsets of dendritic cells (DCs) actively deplete tryptophan within the tumor microenvironment (TME) through upregulation of indoleamine-2,3-dioxygenase 1 (IDO1) or tryptophan-2,3-dioxygenase (TDO2) [117]. Consequently, local tryptophan depletion suppresses anti-tumor immunity by impairing CD8^+^ T cell cytotoxicity while promoting Foxp3 expression, regulatory T cell differentiation, and T cell dysfunction [118,119]. Thus, tryptophan catabolism suppresses immunity through both substrate depletion and metabolite accumulation, although its dominant immunoregulatory effects are largely mediated by kynurenine.

Intracellular tryptophan is metabolized to N-formylkynurenine by three enzymes—IDO1, IDO2, and TDO2—and subsequently converted to kynurenine by arylformamidase (AFMID) [119]. Given that TDO expression is relatively tissue-restricted and IDO2 possesses low catalytic efficiency, IDO1 is considered the primary rate-limiting enzyme for tryptophan catabolism [120]. Notably, IDO1 is upregulated in a wide variety of human malignancies and correlates with poor clinical outcomes [121]. Correspondingly, elevated kynurenine levels are frequently observed across multiple tumor types [122].

Kynurenine acts as a high-affinity endogenous ligand for the aryl hydrocarbon receptor (AhR), thereby orchestrating broad immunosuppressive programs within the TME [123]. Activation of the Kyn–AhR axis promotes regulatory T cell differentiation, suppresses cytotoxic T cell activity through PD-1 upregulation, impairs dendritic-cell immunogenicity, and reshapes helper T cell differentiation toward an immunosuppressive phenotype [124,125,126].

Beyond signaling, kynurenine can exert direct cytotoxic effects. Exogenous kynurenine induces cell cycle arrest at the G1 phase and inhibits the proliferation of activated T cells and NK cells, while sparing resting cells [127]. Another study demonstrated that this anti-proliferative effect is concentration-dependent [128]. Furthermore, kynurenine has been reported to induce T cell apoptosis via Caspase-8 activation and mitochondrial cytochrome c release, independent of Fas/FasL interactions [129]. However, it is important to note that the physiological relevance of this apoptotic pathway is debated, as kynurenine concentrations within the TME are often far lower than those required to induce apoptosis in vitro, suggesting that its in vivo impact may be less pronounced than initially hypothesized [130]. However, the physiological relevance of these direct cytotoxic effects remains controversial, as the kynurenine concentrations required to induce apoptosis in vitro are substantially higher than those typically detected within the TME [131]. This discrepancy suggests that kynurenine-mediated immune suppression in vivo is more likely to depend on sustained metabolic signaling through AhR than on direct cytotoxicity.

#### 2.2.5. Methionine: The Core of Tumor-Mediated Epigenetic Silencing in T Cells

Methionine is an essential amino acid obtained from dietary sources, although it can be regenerated from homocysteine through the methionine cycle using 5-methyltetrahydrofolate or betaine as methyl donors [132].

Beyond its role as a critical substrate for protein synthesis, methionine serves as the sole precursor for S-adenosylmethionine (SAM). As the universal biological methyl donor, SAM drives the methylation of DNA, RNA, histones, and diverse functional proteins, thereby constituting the metabolic foundation of epigenetic regulation [131]. Activated T cells necessitate enhanced methionine uptake to support the protein synthesis and dynamic methylation patterns required for rapid proliferation [133]. However, tumor cells aggressively compete for methionine resources by overexpressing the high-affinity transporter SLC43A2, creating a local “methionine sink” within the TME. This competitive sequestration drastically reduces intracellular methionine and SAM levels in T cells [134]. This metabolic imbalance precipitates the specific loss of histone H3 lysine 79 dimethylation (H3K79me2). As this epigenetic mark is essential for maintaining STAT5 expression, its loss abrogates the STAT5/IL-2 signaling axis, leading to impaired T cell survival, functional dysfunction, and ultimately tumor immune evasion [135]. These findings illustrate how nutrient competition can be directly translated into epigenetic dysfunction, providing a mechanistic link between tumor metabolism and T cell exhaustion.

**Figure 2:**
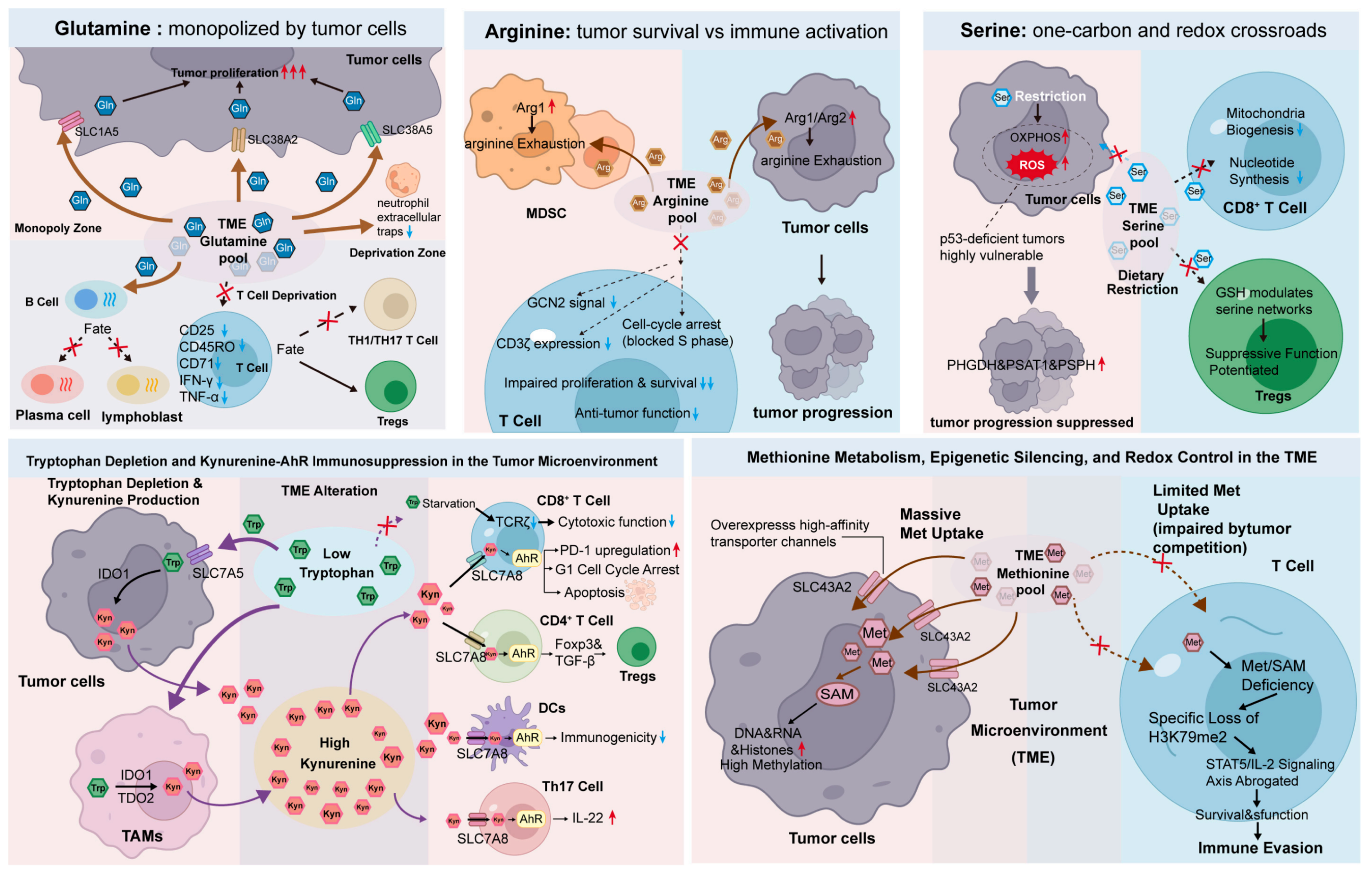
**The amino acid wasteland: metabolic competition and immune subversion in the TME.** Tumor cells aggressively sequester essential amino acids, imposing severe metabolic, bioenergetic, and epigenetic constraints on infiltrating immune cells. **Glutamine:** Malignant cells monopolize glutamine via upregulated SLC1A5 and SLC38A2 transporters. This deprivation suppresses T cell activation markers (CD25, CD45RO, and CD71) and cytokines, skewing differentiation from Th1/Th17 toward a Treg phenotype, while impairing B cell and neutrophil functions. **Arginine:** Elevated ARG1/ARG2 expression by tumor cells and MDSCs exhausts extracellular arginine. This depletion triggers T cell GCN2 signaling, downregulating the TCR CD3ζ chain and inducing G0/G1 cell cycle arrest to halt proliferation. **Serine:** Dietary serine restriction suppresses p53-deficient tumors via oxidative stress but collaterally damages CD8^+^ T cells by impeding nucleotide synthesis. Paradoxically, this depletion potentiates Treg suppressive function via glutathione (GSH) adaptations. **Tryptophan & Kynurenine:** Tumor cells and TAMs use IDO1/TDO2 to metabolize tryptophan into kynurenine. Tryptophan starvation impairs CD8^+^ T cell cytotoxicity, while accumulated kynurenine activates the AhR receptor via SLC7A8. This axis drives immunosuppression by upregulating PD-1, promoting Treg differentiation, and dampening DC immunogenicity. **Methionine:** Tumor cells overexpress SLC43A2 to sequester methionine, fueling SAM generation for hypermethylation. The resulting T cell methionine/SAM deficiency causes the specific loss of histone H3K79me2, abrogating STAT5/IL-2 signaling and leading to profound T cell dysfunction.

Methionine metabolism also contributes to redox homeostasis through the transsulfuration pathway, in which homocysteine is converted into cysteine for the synthesis of glutathione (GSH) and other sulfur-containing metabolites [131,136]. As a major intracellular antioxidant, GSH limits reactive oxygen species (ROS) accumulation and supports T cell activation, proliferation, and survival under metabolic stress [137]. Methionine supplementation has been proven effective in restoring T cell function in tumor-bearing models and cancer patients [134]. Consistently, exogenous SAM significantly potentiates the anti-tumor efficacy of immune checkpoint blockade, suggesting that targeting methionine metabolism represents a novel immune-sensitizing strategy to reverse T cell exhaustion [138]. However, because methionine is also required for tumor-cell proliferation and biosynthesis, systemic supplementation may produce competing effects on malignant and immune cells. Thus, the therapeutic value of targeting methionine metabolism may depend on selectively restoring methionine–SAM signaling in T cells rather than globally increasing methionine availability (Figure 2).

### 2.3. Lipid Architecture in the TME: Shielding Malignancy and Driving Immune Dysfunction

Lipids are essential for tumor-cell survival and expansion, serving as bioenergetic substrates, structural components of cellular membranes, and signaling molecules that regulate proliferation and migration [139]. Accordingly, dysregulated lipid metabolism has emerged as an important feature of cancer progression [140].

To meet their high lipid demands, tumor cells rely on both exogenous uptake and de novo synthesis. They frequently upregulate lipid transporters, including CD36, fatty acid transport proteins (FATPs/SLC27), and fatty acid-binding proteins (FABPs), thereby enhancing lipid acquisition from the tumor microenvironment (TME) [141]. Increased expression of these transporters is associated with aggressive tumor phenotypes and poor clinical outcomes [142]. Tumor cells can also stimulate lipolysis in neighboring adipocytes, increasing the local availability of free fatty acids (FFAs) [143]. Once internalized, fatty acids are transported into mitochondria through carnitine palmitoyltransferase 1A (CPT1A) for fatty acid oxidation (FAO), a pathway frequently enhanced in cancer cells [144].

Tumor cells additionally maintain substantial de novo lipogenic capacity, commonly through upregulation of fatty acid synthase (FASN) [139]. Nevertheless, endogenous synthesis may be insufficient to support rapid tumor expansion, and exogenous lipid uptake can become a major source of membrane and metabolic substrates [145,146]. Because lipid availability varies according to tissue composition, adipocyte abundance, and dietary status, the lipid landscape of the TME is likely to differ substantially across tumor types. This spatial and nutritional heterogeneity may partly explain why lipid-targeted interventions produce inconsistent effects across experimental models and patient populations.

The effects of TME-derived lipids on anti-tumor immune cells are highly context-dependent. Elevated fatty-acid availability can impair CD8^+^ T cell infiltration and cytotoxicity, as observed in breast cancer and obesity-associated tumor models [147,148]. Mechanistically, inhibiting fatty acid oxidation (FAO) can potentiate glycolytic capacity in CD8^+^ T cells, thereby restoring their anti-tumor efficacy [149]. Conversely, another study demonstrated that under conditions of oxygen and glucose deprivation (hypoxia/hypoglycemia), tumor-infiltrating lymphocytes (TILs) can partially preserve CD8^+^ T cell effector function by scavenging and catabolizing fatty acids [150]. Moreover, promoting FAO in CD8^+^ T cells not only expands the population of tumor-reactive CD8^+^ T cells but also potentiates the therapeutic efficacy of PD-1 blockade [151]. These apparently conflicting findings suggest that the consequences of FAO depend on nutrient availability, lipid species, and the metabolic state of the responding T cell rather than on fatty-acid utilization alone.

However, the intracellular accumulation of lipids generally exerts deleterious effects on CD8^+^ T cell function. Tumor-infiltrating CD8^+^ T cells expressing high levels of CD36 preferentially scavenge fatty acids and oxidized low-density lipoproteins (oxLDL); the subsequent accumulation of these oxidized lipids induces T cell functional impairment [152]. Furthermore, in pancreatic cancer, the downregulation of very-long-chain acyl-CoA dehydrogenase (ACADVL) leads to the progressive accumulation of specific long-chain fatty acids in infiltrating CD8^+^ T cells. This accumulation compromises mitochondrial function, ultimately precipitating T cell exhaustion and defective anti-tumor responses [153]. Lipid accumulation also compromises antigen presentation by dendritic cells and suppresses NK-cell glycolysis, granzyme B production, and IFN-γ secretion [154,155].

**Figure 3:**
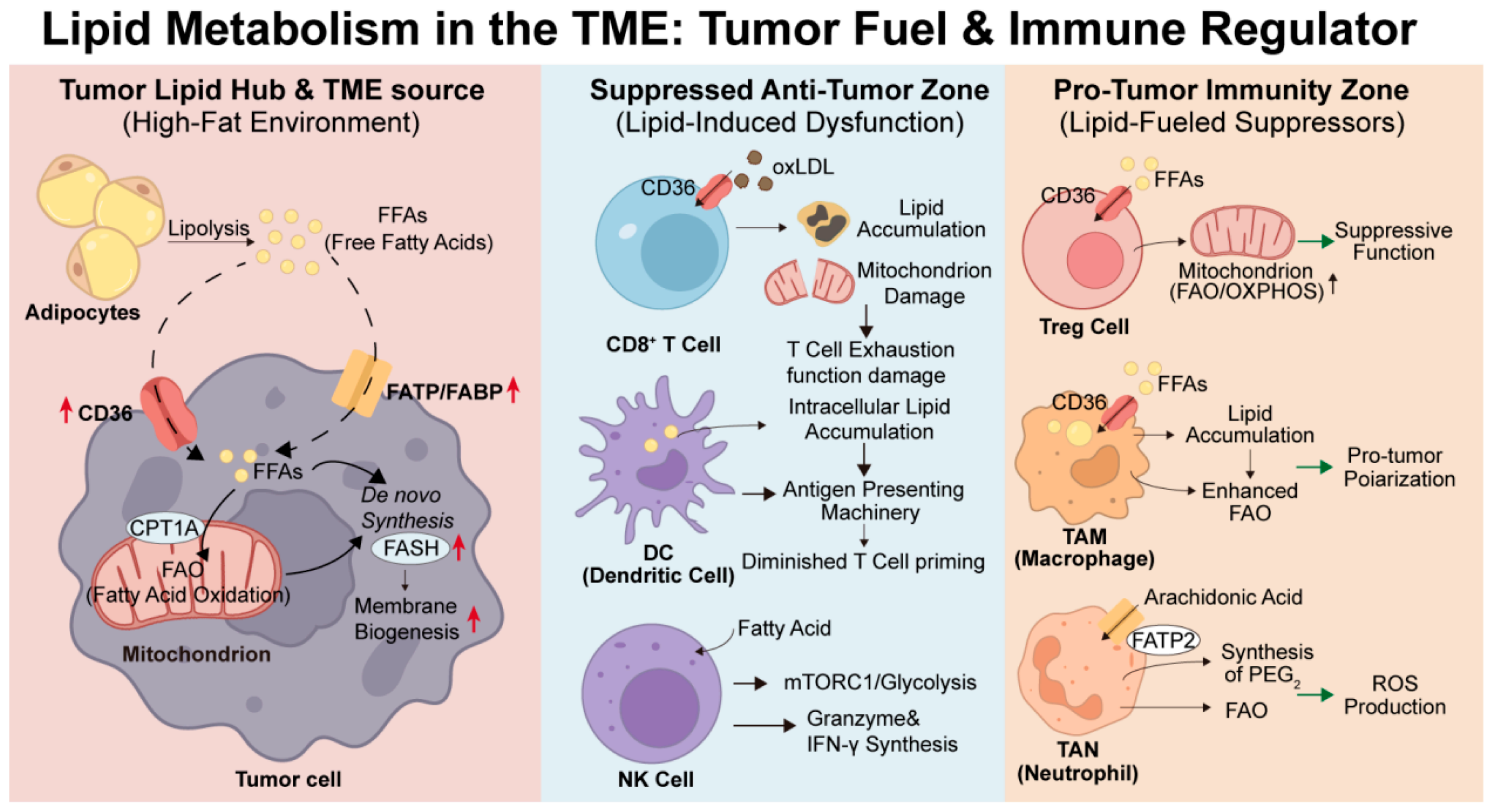
**Lipid architecture in the tumor microenvironment: fueling malignancy and orchestrating immune dysfunction.** The TME lipid landscape, heavily shaped by adipocyte lipolysis and tumor cell metabolic reprogramming, exerts diametrically opposed effects on distinct immune subsets: inducing profound lipotoxicity in anti-tumor effector cells while serving as a potent metabolic fuel for immunosuppressive lineages. **Tumor Lipid Hub (Left)**: Malignant cells aggressively acquire lipids to sustain rapid proliferation and membrane biogenesis. This is achieved through both upregulated de novo lipogenesis (catalyzed by FASN) and the enhanced exogenous scavenging of free fatty acids (FFAs)—often released via adipocyte lipolysis—through membrane transporters including CD36 and FATP/FABP. Internalized FFAs are preferentially channeled into mitochondrial fatty acid oxidation (FAO) via CPT1A to meet immense bioenergetic demands. **Suppressed Anti-Tumor Zone (Middle):** Aberrant lipid accumulation induces severe metabolic paralysis in effector immune cells. In CD8^+^ T cells, CD36-mediated uptake of oxidized low-density lipoproteins (oxLDL) triggers intracellular lipid accumulation and mitochondrial damage, precipitating terminal exhaustion and functional collapse. Similarly, excessive lipid droplet accumulation in dendritic cells (DCs) impairs their antigen-presenting machinery, severely diminishing naive T cell priming. In NK cells, lipid influx blunts mTORC1-driven glycolysis, suppressing the synthesis of critical cytotoxic molecules such as Granzyme B and IFN-γ. **Pro-Tumor Immunity Zone (Right):** Conversely, immunosuppressive subsets exploit TME lipids to fortify their survival and function. Regulatory T cells (Tregs) and tumor-associated macrophages (TAMs) upregulate CD36 to vigorously scavenge FFAs, heavily relying on enhanced mitochondrial FAO and OXPHOS to sustain their suppressive identities and pro-tumor polarization. Concurrently, tumor-associated neutrophils (TANs) upregulate FATP2 to import arachidonic acid, which not only fuels FAO-dependent ROS production but also drives the synthesis of prostaglandin E2 (PGE2), further cementing the immunosuppressive metabolic barrier.

In stark contrast, for immunosuppressive cell subsets, fatty acids within the TME serve as a valuable metabolic fuel. Regulatory T cells (Tregs) upregulate CD36 to enhance fatty acid uptake, exhibiting a metabolic profile characterized by elevated oxidative phosphorylation (OXPHOS) and fatty acid oxidation (FAO). Notably, inhibiting FAO is sufficient to reduce intratumoral Treg abundance and abrogate their suppressive function [156]. Similarly, tumor-associated macrophages (TAMs) express high levels of the scavenger receptor CD36, leading to lipid accumulation and enhanced FAO, which drives their polarization toward a pro-tumor phenotype [157]. Tumor-associated neutrophils (TANs) increase FATP2-mediated arachidonic-acid uptake and prostaglandin E2 (PGE2) synthesis, while enhanced FAO under glucose restriction promotes reactive oxygen species (ROS) production and T cell suppression [158,159] (Figure 3). Thus, lipid metabolism creates a selective advantage for immunosuppressive populations while producing variable effects on effector lymphocytes. This cell-type specificity represents a central challenge for lipid-targeted therapy, because global inhibition of lipid uptake or FAO may suppress tumor-supporting immune cells but also eliminate metabolic adaptations required by CD8^+^ T cells in nutrient-poor regions of the TME (Table 1).

## 3. Metabolic Crosstalk and Network Regulation Within the Tumor Microenvironment

Although carbohydrate, amino acid, and lipid metabolism are conventionally discussed as independent metabolic programs, accumulating evidence indicates that these pathways are highly interconnected within the tumor microenvironment (TME) [2,11]. Rather than functioning as isolated nutrient sources, metabolites are dynamically exchanged among tumor cells, stromal cells, and immune cells and are extensively integrated through shared metabolic intermediates, signaling pathways, and epigenetic regulation [160]. Consequently, alterations in one metabolic pathway frequently induce compensatory changes in others, ultimately reshaping immune-cell differentiation, effector function, and therapeutic responsiveness. This metabolic crosstalk not only underlies the remarkable plasticity of tumor metabolism but also partly explains why targeting a single metabolite often produces limited or heterogeneous therapeutic responses [5]. Therefore, understanding tumor immunometabolism from a network perspective, rather than focusing on individual metabolites in isolation, is becoming increasingly important for developing effective metabolic immunotherapies.

A notable feature of metabolic crosstalk is that seemingly distinct nutrients frequently converge on a limited number of shared metabolic intermediates [11]. For example, glucose and glutamine both replenish the tricarboxylic acid (TCA) cycle and contribute to acetyl-CoA production, thereby influencing lipid biosynthesis as well as histone acetylation [87]. Likewise, serine metabolism is closely coupled to the methionine cycle through one-carbon metabolism, providing methyl groups required for the generation of S-adenosylmethionine (SAM), the universal methyl donor for DNA and histone methylation. In parallel, glutamine-derived α-ketoglutarate (α-KG), together with succinate, fumarate, and 2-hydroxyglutarate (2HG), regulates the activity of α-KG-dependent dioxygenases, thereby coordinating epigenetic remodeling and immune-cell differentiation. These observations indicate that multiple metabolic pathways ultimately converge to regulate common cellular processes, including bioenergetics, redox homeostasis, and epigenetic programming.

Metabolic crosstalk within the TME extends beyond intracellular metabolic rewiring and is fundamentally driven by continuous metabolite exchange among tumor cells, stromal cells, and immune cells [25]. Rather than acting as passive bystanders, these cellular populations actively reshape the extracellular metabolic landscape through nutrient consumption, metabolite secretion, and reciprocal metabolic adaptation. Tumor cells consume large quantities of glucose, glutamine, methionine, and arginine, thereby limiting nutrient availability for infiltrating lymphocytes, while simultaneously releasing metabolites such as lactate, succinate, fumarate, and kynurenine that further suppress anti-tumor immunity [75,141]. In parallel, stromal cells actively participate in this metabolic network. For example, cancer-associated fibroblasts (CAFs) secrete lactate that can be reutilized by tumor cells through the reverse Warburg effect, whereas tumor-associated macrophages (TAMs) produce metabolites including itaconate and succinate that profoundly influence T cell activation and macrophage polarization. Likewise, adipocytes release free fatty acids that support tumor growth while simultaneously reshaping the metabolic programs of T cells, macrophages, and neutrophils. Therefore, the metabolic landscape of the TME should be regarded as a dynamic ecosystem in which nutrients and metabolites function not only as metabolic substrates but also as mediators of intercellular communication.

The extensive metabolic crosstalk within the TME also provides an important explanation for the limited efficacy of many single-target metabolic interventions. Because glucose, amino acid, and lipid metabolism are tightly interconnected, modulation of one metabolic pathway frequently induces compensatory activation of others. Likewise, many metabolites exert pleiotropic and context-dependent effects that vary according to nutrient availability, cellular differentiation, and tissue localization. For example, lactate suppresses effector T cell function while simultaneously serving as an energy source for regulatory T cells, whereas intracellular and extracellular succinate exhibit distinct immunological functions. These observations suggest that therapeutic strategies focusing on a single metabolite or enzyme may fail to fully remodel the metabolic ecosystem of the TME. Instead, future metabolic immunotherapies may benefit from integrating multiple metabolic targets while simultaneously considering cell-specific metabolic requirements and dynamic metabolic adaptation.

**Figure 4:**
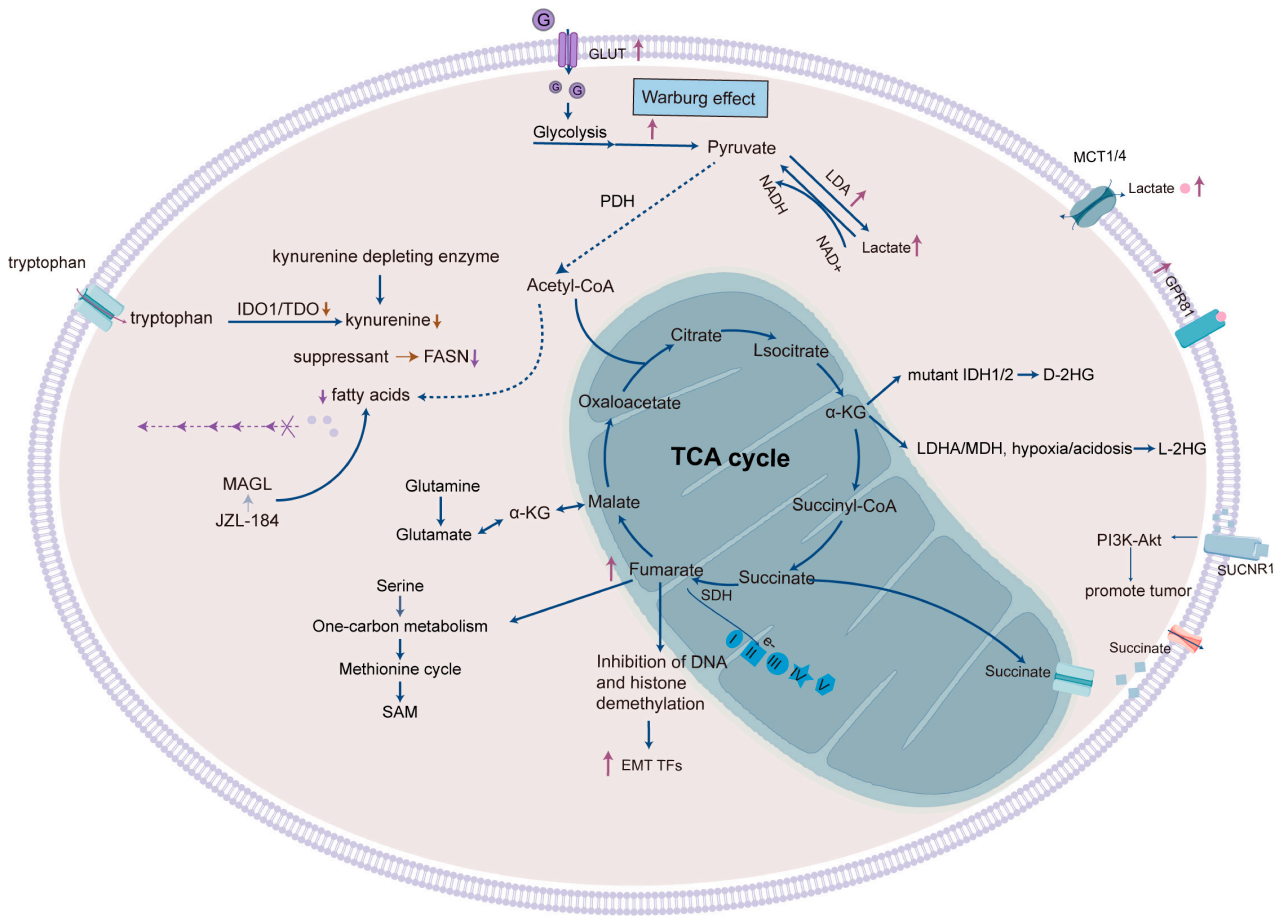
**Integrated metabolic reprogramming and metabolite signaling in tumor cells**.

Metabolic regulation within the TME should therefore be understood as an integrated and dynamic network rather than a collection of independent pathways. This systems-level perspective provides the conceptual basis for the metabolism-targeted interventions discussed in the following section (Figure 4).

Glucose is imported through GLUT1 and metabolized by glycolysis to generate pyruvate. Pyruvate is subsequently converted either to lactate by lactate dehydrogenase A (LDHA) or to acetyl-CoA through the pyruvate dehydrogenase complex, thereby linking glycolysis to the tricarboxylic acid (TCA) cycle. Lactate can be exported through monocarboxylate transporters and activate GPR81-dependent signaling to support tumor adaptation and progression. Glutamine is converted to glutamate and subsequently to α-ketoglutarate (α-KG), replenishing the TCA cycle and supporting biosynthetic and epigenetic programs. Serine metabolism contributes to one-carbon metabolism and the methionine cycle, thereby regulating S-adenosylmethionine (SAM) availability and methylation reactions. TCA-cycle dysfunction results in the accumulation of oncometabolites, including succinate, fumarate, and 2-hydroxyglutarate (2HG). Succinate can activate SUCNR1-mediated signaling, whereas succinate, fumarate, and D-2HG inhibit α-KG-dependent dioxygenases, leading to impaired DNA and histone demethylation and extensive epigenetic remodeling. Tryptophan catabolism through IDO1 or TDO2 generates kynurenine, while lipid availability is regulated through fatty-acid synthesis and monoacylglycerol lipase (MAGL)-dependent lipid mobilization. Together, these interconnected pathways illustrate how carbohydrate, amino-acid, and lipid metabolism converge on shared metabolic intermediates and signaling mechanisms to promote tumor progression.

## 4. Translational Strategies: Dismantling the Metabolic Shield for Cancer Immunotherapy

In recent years, cancer immunotherapies, represented by immune checkpoint blockade (ICB, e.g., anti-PD-1/PD-L1 antibodies) and adoptive cell therapy (ACT, e.g., CAR-T), have achieved breakthrough advances in selected solid and hematological malignancies. However, in clinical practice, a significant proportion of patients—often exceeding 50%—either fail to respond or rapidly develop acquired resistance to these regimens. This therapeutic ceiling may be largely attributed to the nutrient-deprived landscape faced by tumor-infiltrating T cells (TILs) within the tumor microenvironment (TME). The scarcity of essential nutrients, such as glucose and glutamine, imposes severe bioenergetic restrictions on TILs, rendering them unable to sustain the metabolic demands required for clonal expansion and the biosynthesis of effector molecules (e.g., cytokines and granzymes). Concurrently, the accumulation of oncometabolites—such as lactate, succinate, and kynurenine—impairs T cell expansion and function via epigenetic reprogramming or direct metabolic interference. These factors induce a T cell exhaustion phenotype while fostering the differentiation of immunosuppressive subsets, including tumor-associated macrophages (TAMs), myeloid-derived suppressor cells (MDSCs), and regulatory T cells (Tregs). This distinct state of metabolic anergy, coupled with the establishment of an immunosuppressive metabolic network, likely constitutes the core bottleneck limiting the clinical efficacy of T cell-centric immunotherapies. To surmount this hurdle, researchers are actively developing metabolic modulation strategies. Current interventions primarily focus on two avenues: (1) modulating the production and clearance of immunosuppressive metabolites and (2) enhancing the metabolic fitness of T cells. The specific therapeutic interventions emerging from these paradigms are discussed below.

### 4.1. Targeting Oncometabolite Production, Transport Bridges, and Receptor Nodes

As detailed in the preceding sections, metabolites such as lactate, succinate, fumarate, 2HG, itaconate, kynurenine, and fatty acids accumulate significantly within the tumor microenvironment (TME). These molecules exert potent immunosuppressive effects via distinct pathways, thereby constraining the anti-tumor activity of T cells. To abrogate these deleterious effects, therapeutic interventions can be strategically deployed across four critical “nodes”: (1) The Origin, targeting the biosynthetic enzymes and substrate availability responsible for the generation of immunosuppressive metabolites; (2) The Transport Bridge, modulating the transport proteins governing both the efflux of metabolites from tumor cells into the extracellular space and their subsequent influx into T cells; (3) Signaling Receptors, blockading the interaction between metabolites and their cognate immunosuppressive receptors; and (4) The Metabolites Themselves, directly neutralizing or enhancing the systemic/local clearance of the accumulated metabolites. The following discussion will dissect specific targeting strategies for the aforementioned oncometabolites, structured around these four intervention points.

**Lactate:** Lactate is generated via the reduction of pyruvate catalyzed by LDHA/B and shuttled across cellular membranes through MCT1/4. Additionally, it mediates signal transduction by activating GPR81 or GPR132. Recent years have witnessed significant strides in targeting lactate metabolism, with several agents advancing to Phase I/II clinical trials. Regarding LDHA/B targeting, diverse strategies have been developed and validated. For instance, specific LDHA inhibitors—including AT-101, GSK2837808A, and GNE-140—effectively abrogate lactate generation by directly inhibiting enzymatic activity [161,162]. Furthermore, long non-coding RNAs (e.g., LncRNA GLTC) and metabolic analogs such as oxalate have also demonstrated inhibitory effects on LDHA/B [163,164]. Notably, AT-101 has exhibited therapeutic efficacy in clinical trials involving patients with advanced lung and prostate cancer [165]. In parallel, the MCT1/2 inhibitor AZD3965 has completed Phase I/II trials in patients with diffuse large B-cell lymphoma (DLBCL) and Burkitt lymphoma, yielding promising clinical outcomes [166]. By comparison, research targeting GPR81 remains nascent; although specific antagonists have been identified, their in vitro and in vivo efficacy warrants further verification [167]. A novel paradigm involves repurposing lactate as a fuel for CD8^+^ T cells; specifically, Lithium Carbonate facilitates the mitochondrial translocation of MCT1, promoting intra-mitochondrial lactate metabolism by LDHB to generate energy, thereby enhancing the activation and anti-tumor effector function of tumor-reactive CD8^+^ T cells [168]. Additionally, combinatorial strategies targeting multiple nodes have shown potential. For example, nanoparticles loaded with α-cyano-4-hydroxycinnamate (CHC) and lactate oxidase (LOX) are designed to degrade within the acidic microenvironment to release their payload: CHC inhibits MCT1 expression to block lactate uptake, while LOX catalyzes lactate decomposition, achieving a synergistic dual inhibition of tumor growth [169].

**Succinate:** Succinate generation primarily relies on the reaction catalyzed by succinyl-CoA synthetase converting succinyl-CoA, whereas its accumulation is predominantly associated with SDH inhibition. As previously delineated, succinate is exported from the mitochondria via SLC25A10 and specific monocarboxylate or dicarboxylate transporters to the extracellular space, where it activates GPR91 (SUCNR1) [41]. Although extracellular succinate can enter cells via SLC13A2 and SLC13A3, it is noteworthy that these transporters are predominantly expressed in the kidney and intestine [170]. Therapeutic strategies targeting the succinate metabolic pathway have garnered increasing attention but remain in the nascent stages of exploration. Given the pivotal role of SUCNR1 in the pathology of various diseases (e.g., hypertension, diabetes, and myocardial ischemia–reperfusion injury), early research focused on developing small-molecule SUCNR1 antagonists [171]; however, the widespread distribution of SUCNR1 in the liver, kidney, and gastrointestinal tract implies that systemic inhibition could precipitate unpredictable adverse effects, thereby impeding the clinical translation of SUCNR1-based drugs [33]. To surmount this hurdle, we engineered microparticles overexpressing succinate receptors capable of specifically scavenging succinate from the TME and delivering it into tumor-associated macrophages (TAMs). The resulting intracellular accumulation successfully induced the repolarization of TAMs from a pro-tumor to an anti-tumor phenotype. This strategy provides a novel paradigm for targeted succinate therapy in the TME and opens potential avenues for precision interventions based on succinate metabolism.

**Fumarate:** Fumarate is produced via the oxidation of succinate catalyzed by SDH, and its aberrant accumulation is primarily linked to functional deficiencies in fumarate hydratase (FH). Similar to other intermediates, fumarate is transported via monocarboxylate and dicarboxylate transporters, although a specific receptor remains unreported. Current therapeutic efforts targeting fumarate metabolism principally focus on FH-deficient renal cell carcinoma (RCC), aiming to induce direct tumor cytotoxicity by exploiting metabolic vulnerabilities. In FH-deficient cells, the massive accumulation of fumarate significantly depletes glutathione, leading to elevated intracellular reactive oxygen species (ROS) levels. Research indicates that blocking the glycolytic pathway with LDHA inhibitors further exacerbates ROS accumulation, thereby driving tumor cell apoptosis. Furthermore, FH-deficient cells exhibit a dependency on heme oxygenase (HO) for glutamine metabolism and bilirubin excretion; consequently, selective HO inhibition (e.g., using zinc protoporphyrin IX) results in tumor-specific lethality [172].

**2-Hydroxyglutarate (2HG):** The generation of D-2HG and L-2HG is catalyzed by mutant IDH1/2 and the promiscuous activity of LDH/MDH under metabolically dysregulated conditions, respectively. Significant strides have been made in therapeutic strategies targeting IDH1 mutations to inhibit D-2HG production. Notably, IDH1 inhibitors have demonstrated substantial efficacy in Phase III clinical trials and have been FDA-approved for the treatment of relapsed or refractory IDH1-mutant acute myeloid leukemia (AML) [173]. The administration of IDH1 inhibitors, such as Vorasidenib and Ivosidenib, effectively reduces D-2HG levels in IDH1-mutant gliomas and correlates with signs of immune activation [174]. Beyond small molecules, a novel strategy involves genetically engineering CAR-T cells to overexpress enzymes capable of metabolizing D-2HG. These engineered CAR-T cells convert D-2HG into α-KG, thereby boosting oxidative metabolism and cytokine production, which significantly potentiates CAR-T cell-mediated cytotoxicity against cancer cells [175].

**Itaconate:** Itaconate is generated via the decarboxylation of cis-aconitate catalyzed by ACOD1 (also known as IRG1); however, its intracellular transport mechanisms and cognate receptors remain incompletely defined. The appreciation of itaconate as a pivotal player in the tumor microenvironment is relatively recent, and research into targeting this metabolic pathway is still in its infancy. Currently, several potential strategies for the targeted modulation of ACOD1 have been proposed: one approach involves increasing intracellular citrate levels to compete with cis-aconitate for ACOD1 binding, thereby inhibiting itaconate synthesis [176]; another utilizes microRNA-144 (miR-144) to target and suppress ACOD1 expression, consequently lowering itaconate levels [177]. These strategies offer potential research avenues and therapeutic possibilities for modulating the role of itaconate in inflammation and the tumor microenvironment.

**Kynurenine:** Kynurenine is generated from tryptophan via catalysis by IDO or TDO. Currently, a diverse array of small-molecule inhibitors targeting IDO1 and TDO have been developed and have advanced to clinical trials [116]. Furthermore, dual inhibitors capable of simultaneously targeting both IDO1 and TDO have entered clinical evaluation as monotherapies for solid tumors, demonstrating promising anti-tumor efficacy [178]. An alternative strategy employs the systemic administration of kynurenine-degrading enzymes (e.g., kynureninase), which catabolize kynurenine into metabolites that are immunologically inert, non-toxic, and readily cleared, thereby suppressing tumor growth. Studies indicate that the deployment of kynurenine-degrading enzymes significantly augments the infiltration of CD8^+^ T cells within the TME and enhances anti-tumor immune responses [179].

**Figure 5:**
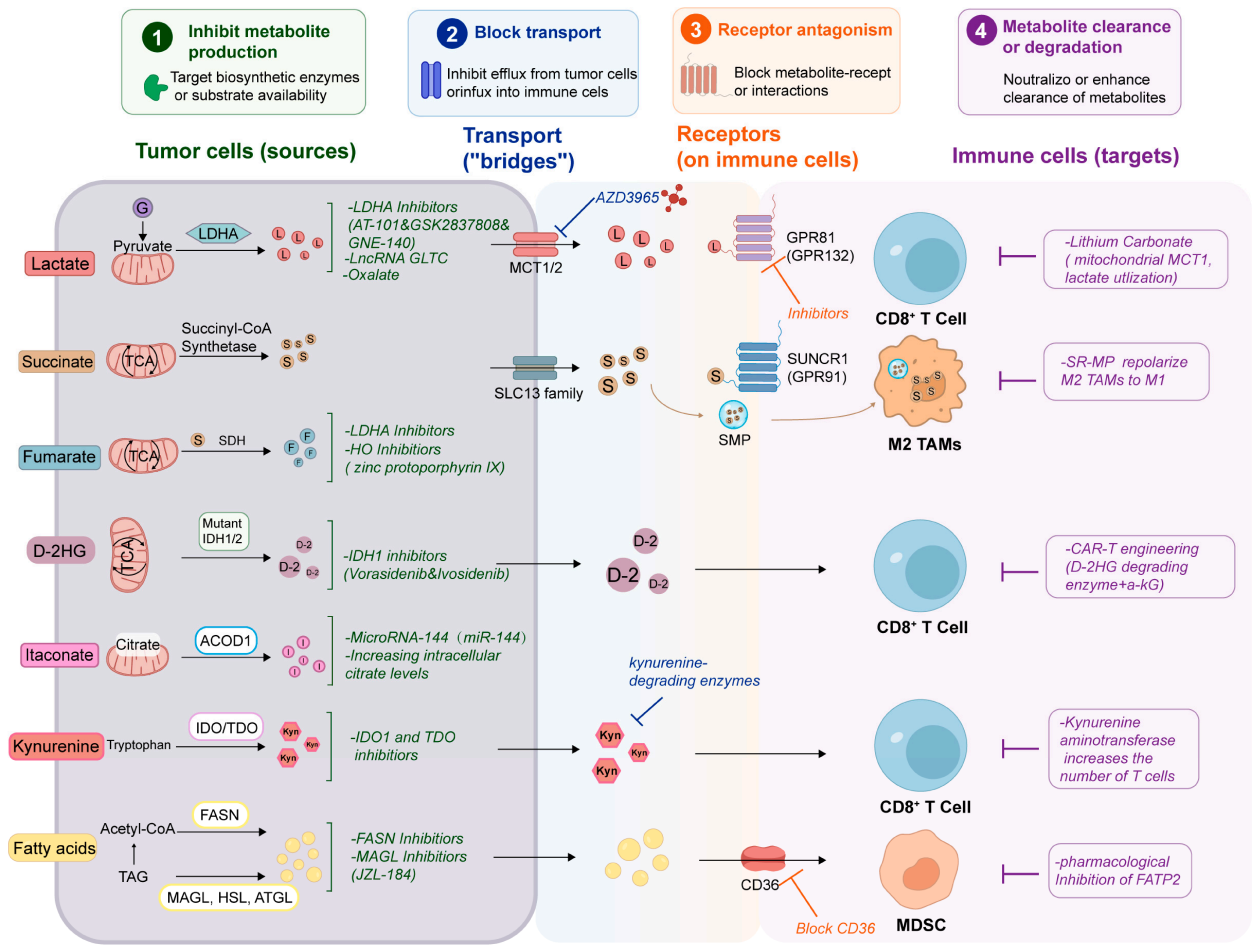
**Therapeutic interventions to dismantle the immunosuppressive metabolic shield in the TME.** To overcome metabolic barriers and reinvigorate anti-tumor immunity, current pharmacological and engineered strategies are strategically deployed across four critical intervention nodes: **(1) Inhibit metabolite production:** Targeting key biosynthetic enzymes within tumor cells to halt the initial generation of oncometabolites. Representative strategies include LDHA inhibitors (e.g., AT-101 and GSK2837808A) to restrict lactate synthesis, mutant IDH1 inhibitors (e.g., Vorasidenib and Ivosidenib) to suppress D-2HG, IDO/TDO inhibitors to block kynurenine production, and FASN/MAGL inhibitors (e.g., JZL-184) to constrain fatty acid availability. **(2) Block transport (“bridges”):** Severing the metabolic crosstalk by inhibiting the efflux from tumor cells or the influx into immune cells. A prominent example is the disruption of the lactate shuttle via MCT1/2 blockade (e.g., AZD3965). **(3) Receptor antagonism:** Preventing extracellular metabolites from triggering downstream immunosuppressive cascades by blocking their cognate receptors on immune cells. Potential targets include GPR81/GPR132 for lactate, SUCNR1 (GPR91) for succinate, and the scavenger receptor CD36 for lipid uptake. **(4) Metabolite clearance or degradation:** Directly neutralizing accumulated TME waste or rewiring immune cells to repurpose these metabolites. Innovative approaches include administering systemic kynurenine-degrading enzymes, engineering CAR-T cells to co-express D-2HG-degrading enzymes, deploying succinate-receptor microparticles (SR-MP) to scavenge succinate and repolarize M2 TAMs, and utilizing Lithium Carbonate to redirect lactate toward mitochondrial utilization in CD8^+^ T cells.

**Fatty acids:** The biological pool of fatty acids relies primarily on the de novo synthesis pathway from acetyl-CoA mediated by FASN, as well as the lipolysis of triglycerides catalyzed by hormone-sensitive lipase (HSL) and adipose triglyceride lipase (ATGL); the released free fatty acids traverse membranes via transporters such as CD36 and the fatty acid transport protein (FATP) family. FASN, as the rate-limiting enzyme in fatty acid biosynthesis, represents a potent therapeutic target in various malignancies; its inhibitors significantly trigger tumor cell apoptosis by perturbing membrane phospholipid homeostasis and suppressing pro-survival signaling pathways, a potential confirmed by preclinical studies and early-phase clinical trials [180,181]. Additionally, the aberrant overexpression of monoacylglycerol lipase (MAGL) in tumor cells contributes to elevated intracellular free fatty acids, whereas the specific inhibitor JZL-184 effectively lowers fatty acid levels by blocking MAGL-mediated monoacylglycerol hydrolysis, thereby inhibiting tumor growth [182]. Within the TME, CD36-mediated fatty acid uptake in tumor-infiltrating CD8^+^ T cells elevates intracellular lipid peroxidation, thereby impairing cytotoxic cytokine secretion and anti-tumor immunity via a ferroptosis mechanism. Blocking CD36 effectively reverses this dysfunction and yields synergistic anti-tumor effects when combined with anti-PD-1 antibodies [183]. In parallel, pharmacological inhibition of FATP2 in MDSCs abrogates their immunosuppressive activity by preventing lipid accumulation, significantly delaying tumor progression and exhibiting superior tumor control in murine models when combined with immune checkpoint inhibitors [158] (Figure 5, Table 2).

### 4.2. Enhancing T Cell Metabolic Competitiveness and Resilience

Given that tumor cells and immunosuppressive subsets possess a significantly superior capacity for sequestering nutrients like glucose and glutamine compared to T cells, infiltrating T cells often remain metabolically undersupplied even under conditions of dietary nutrient supplementation [11]. To circumvent this bottleneck, recent research has pivoted toward engineering T cells with enhanced nutrient acquisition capabilities to survive the “tug-of-war” for resources. The Solute Carrier (SLC) superfamily, comprising over 400 members, is fundamental to the transmembrane transport of diverse metabolites [188]. Specifically, SLC2A1 (GLUT1) serves as the primary glucose transporter in T cells and is indispensable for activation and effector function [189]. Considering the requisite metabolic switch from oxidative phosphorylation to glycolysis during T cell activation, the ectopic overexpression of SLC2A1 represents a promising strategy to improve the metabolic fitness of CAR-T cells within the TME, thereby augmenting their glycolytic flux and effector output [11]. Similarly, tumor cells create a local methionine shortage via high expression of the transporter SLC43A2, effectively outcompeting T cells. Research demonstrates that while SLC43A2-deficient tumors allow for restored T cell responses, the forced expression of SLC43A2 in CAR-T cells bolsters their competitive edge for methionine, thereby promoting survival, functional robustness, and memory differentiation [134]. Beyond the plasma membrane, the SLC25 family located in the inner mitochondrial membrane mediates the exchange of metabolites, nucleotides, and cofactors between the mitochondria and cytosol. For instance, SLC25A1 facilitates the mitochondrial–cytosolic citrate shuttle, which is critical for fatty acid synthesis and acetylation reactions [190]. Since silencing SLC25A1 compromises Th1 proliferation and transcriptional remodeling, its overexpression holds significant potential to potentiate CAR-T therapeutic efficacy [191].

An alternative paradigm focuses on metabolic reprogramming to bolster T cell resilience against the hostile microenvironment. The glycolytic metabolite phosphoenolpyruvate (PEP) is pivotal for sustaining TCR-mediated Ca^2+^-NFAT signaling and effector function; however, glucose deprivation in the TME precipitates a sharp decline in intracellular PEP. To counteract this, the overexpression of PCK1 (phosphoenolpyruvate carboxykinase 1) in adoptively transferred T cells acts as a metabolic bypass, converting oxaloacetate to PEP, thereby sustaining T cell survival and amplifying anti-tumor efficacy [192]. Metabolic pre-conditioning offers another avenue: culturing CAR-T cells under conditions mimicking the nutrient-poor TME—specifically, in low-glutamine media—imparts a memory-like phenotype (“metabolic imprinting”). These cells, having adapted to scarcity, exhibit reduced expression of inhibitory receptors and superior in vivo anti-tumor potency upon adoptive transfer compared to those expanded in conventional media [193].

Furthermore, since therapeutic persistence correlates with superior tumor clearance and patient survival [194], strategies promoting the formation of long-lived memory CD8^+^ T cells—which rely on oxidative phosphorylation (OXPHOS) rather than glycolysis and are thus metabolically distinct from effector cells—are highly advantageous in the glucose-deprived TME [195]. Under glucose starvation, the impairment of the Pentose Phosphate Pathway (PPP) typically depletes NADPH and glutathione, leading to elevated Reactive Oxygen Species (ROS) and exhaustion. Pharmacological inhibition of IDH2 effectively rewires metabolic flux into the PPP, mitigating ROS accumulation and preventing terminal differentiation. Concurrently, the resulting elevation in cytosolic citrate fuels Acetyl-CoA production via ATP-citrate lyase, driving H3K27 acetylation to transcribe memory-associated genes, ultimately potentiating CAR-T efficacy [196]. In a parallel approach, blocking pyruvate entry into mitochondria via genetic ablation or pharmacological inhibition (e.g., UK5099) of the Mitochondrial Pyruvate Carrier (MPC) forces a metabolic shift that drives CD8^+^ T cell differentiation toward a memory phenotype. CAR-T cells pre-treated with UK5099 demonstrate durable anti-tumor activity in both immunocompetent solid tumor models and xenograft leukemia models [197].

In parallel, exogenous arginine supplementation has been identified as a potent driver of T cell metabolic reprogramming. Elevating intracellular arginine concentrations to approximately twice the baseline levels triggers a profound metabolic shift, transitioning T cells from a glycolysis-dominant state toward oxidative phosphorylation (OXPHOS). Although the precise molecular underpinnings driving this broad metabolic alteration remain to be fully elucidated, current evidence suggests that arginine enhances metabolic flux through the tricarboxylic acid (TCA) cycle—potentially via the upregulation of the serine biosynthesis pathway—thereby fueling OXPHOS. Phenotypically, arginine conditioning promotes the differentiation of T cells into a central memory phenotype characterized by high expression of CCR7 and CD62L, conferring superior in vivo persistence and anti-tumor activity in tumor models [100]. Conversely, regarding cellular aging, research highlights that while senescent T cells maintain active glucose metabolism, they exhibit profound dysregulation in lipid metabolism. Tumor cells and Tregs accelerate T cell senescence by inducing the upregulation of Group IVA phospholipase A2 (PLA2G4A), which precipitates maladaptive alterations in lipid metabolism. Pharmacological inhibition of PLA2G4A effectively reprograms effector T cell lipid metabolism, averting senescence during in vitro expansion and significantly potentiating anti-tumor immune responses and immunotherapy efficacy in murine melanoma and breast cancer models [198].

**Figure 6:**
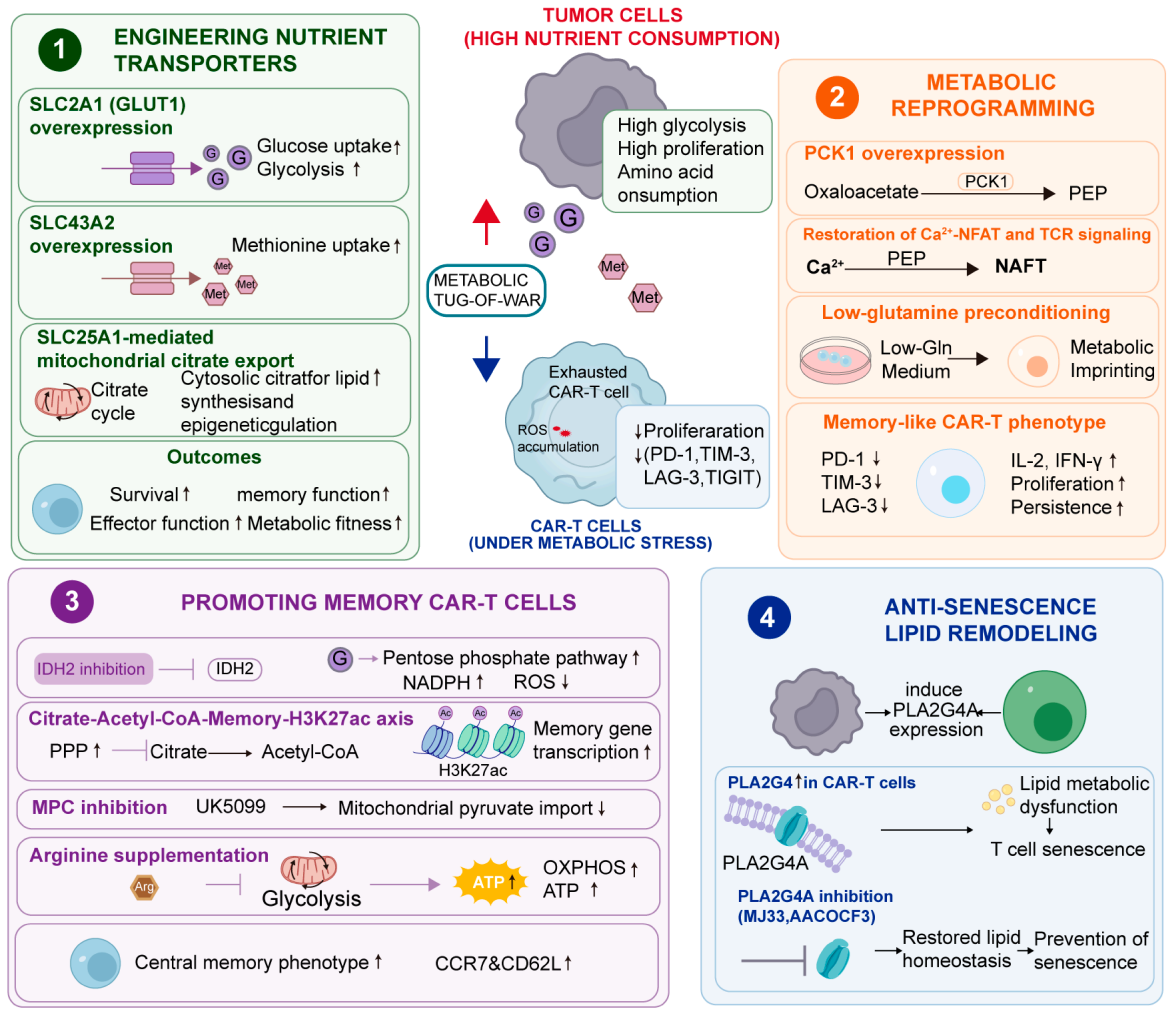
**Metabolic engineering strategies for overcoming metabolic bottlenecks in cancer immunotherapy.** The nutrient-deprived and immunosuppressive tumor microenvironment (TME) restricts the efficacy of T cell-based immunotherapies by limiting nutrient availability and inducing metabolic dysfunction. Representative strategies to overcome these metabolic bottlenecks include (1) engineering nutrient transporters to improve nutrient acquisition; (2) metabolic reprogramming to enhance T cell activation and adaptation to nutrient stress; (3) promoting memory-like CAR-T cells through optimization of glucose, mitochondrial, and amino acid metabolism; and (4) lipid metabolic remodeling to prevent T cell senescence and sustain long-term anti-tumor function. These approaches collectively enhance T cell metabolic fitness and provide promising opportunities for improving the efficacy and durability of cancer immunotherapy.

Together, these approaches enhance the metabolic competitiveness and environmental resilience of adoptively transferred T cells, offering a means to improve their persistence and activity in solid tumors (Figure 6).

### 4.3. Challenges and Future Directions in Metabolic Targeting

Although considerable progress has been made in elucidating how metabolic reprogramming shapes anti-tumor immunity, the clinical translation of metabolism-targeted therapies has been considerably slower than initially anticipated. While numerous metabolic modulators have demonstrated encouraging efficacy in preclinical models, only a limited number have progressed to clinical evaluation, and even fewer have achieved durable clinical benefit [199]. This discrepancy suggests that therapeutic success depends not only on the biological importance of individual metabolic pathways but also on the complexity, redundancy, and dynamic adaptation of metabolic networks within the tumor microenvironment (TME) [2,8,48,160]. Moreover, because metabolic pathways are extensively shared between tumor cells and immune cells, interventions targeting a single metabolite frequently produce both anti-tumor and immunomodulatory effects, complicating therapeutic optimization [2,9]. These challenges underscore the need to view tumor metabolism as an integrated regulatory network rather than a collection of isolated metabolic pathways.

A major challenge in metabolic immunotherapy is the remarkable metabolic plasticity of both tumor cells and immune cells [8]. Rather than relying on a single metabolic pathway, tumor cells rapidly adapt to metabolic stress by rewiring interconnected networks of glucose, amino acid, and lipid metabolism. Likewise, immune cells dynamically adjust their metabolic programs according to nutrient availability and activation status [11]. Consequently, inhibition of a single metabolic pathway often triggers compensatory activation of alternative pathways, thereby attenuating therapeutic efficacy. For example, suppression of glycolysis may enhance fatty acid oxidation or glutamine utilization, whereas nutrient restriction can induce adaptive metabolic reprogramming that supports tumor survival [162,193,200]. These observations indicate that effective metabolic interventions should consider the metabolic network as a whole rather than targeting individual metabolites or enzymes in isolation.

Another major obstacle to clinical translation is the dependence of metabolic interventions on cellular and environmental context. Unlike conventional oncogenic targets, many metabolic pathways are simultaneously shared by tumor cells and immune cells, making selective therapeutic modulation particularly challenging [160]. As discussed throughout this review, the same metabolite may exert distinct—or even opposing—effects depending on the responding cell type, metabolic state, or subcellular localization. For example, lactate suppresses the function of effector T cells while serving as an important metabolic substrate for regulatory T cells [21,28], whereas intracellular and extracellular succinate elicit fundamentally different biological responses [26,46]. Similarly, arginine and methionine are essential for both tumor growth and T cell activation, creating an inherent therapeutic dilemma [100,134]. These examples support cell-selective and state-specific metabolic modulation rather than indiscriminate systemic inhibition or supplementation.

Taken together, these challenges highlight that the next generation of metabolic immunotherapies should move beyond single-metabolite intervention toward integrated and precision-based strategies [2,9]. Rational combinations of metabolic modulators with established immunotherapies, including immune checkpoint blockade and adoptive cell therapy, are likely to provide greater therapeutic benefit than either approach alone [199]. In parallel, biomarker-guided patient stratification will be essential, as metabolic dependencies vary substantially across tumor types, disease stages, and individual patients. Finally, increasing attention should be directed toward understanding metabolic crosstalk among carbohydrate, amino acid, and lipid metabolism, as well as the reciprocal interactions between tumor cells, stromal cells, and immune cells [2,139,201]. A more comprehensive understanding of these dynamic metabolic networks will facilitate the development of safer, more selective, and more effective metabolic interventions for cancer immunotherapy.

Emerging technologies are increasingly enabling the spatial and cell-type-specific resolution of tumor immunometabolism [9]. Single-cell transcriptomic and multi-omics profiling can identify metabolically distinct tumor and immune-cell subsets, whereas spatial metabolomics and imaging mass spectrometry provide information on the regional distribution of nutrients and immunosuppressive metabolites within intact tumors [202,203]. Stable-isotope tracing further enables direct assessment of nutrient utilization and intercellular metabolite exchange in vivo [204]. In parallel, CRISPR-based functional screens can systematically identify metabolic genes that regulate T cell persistence, exhaustion, and resistance to nutrient stress. Integration of these approaches with patient-derived organoids and longitudinal clinical samples may facilitate the discovery of causal metabolic dependencies and predictive biomarkers. Nevertheless, metabolite instability, limited spatial resolution, differences between ex vivo and in vivo metabolic states, and the complexity of integrating multimodal datasets remain important technical challenges.

## 5. Conclusions

It is now well established that metabolic reprogramming within the TME is a major determinant of anti-tumor immunity and therapeutic resistance. Nutrient competition and the accumulation of immunoregulatory metabolites jointly impair the metabolic fitness of effector T cells while supporting tumor cells and immunosuppressive populations. Importantly, carbohydrate, amino acid, and lipid metabolism do not operate as isolated pathways but converge through shared metabolic intermediates, nutrient-sensing pathways, redox regulation, and epigenetic remodeling. The TME should therefore be understood as a dynamic metabolic ecosystem in which tumor cells, stromal cells, and immune cells continuously reshape one another through nutrient consumption, metabolite exchange, and reciprocal adaptation. This network-level organization explains why the biological effects of metabolic perturbation vary substantially across cellular compartments and tumor contexts.

Emerging evidence is expanding the therapeutic focus from suppressing tumor-associated metabolic pathways to strengthening the metabolic resilience of anti-tumor immune cells. In addition to targeting enzymes, transporters, receptors, and accumulated metabolites, recent preclinical strategies have incorporated nutrient-transporter engineering, metabolic preconditioning, memory-cell programming, metabolite-scavenging platforms, and lipid-metabolic remodeling. These approaches suggest that improving nutrient acquisition, metabolic flexibility, persistence, and resistance to exhaustion may be as important as directly restricting tumor metabolism. Nevertheless, several fundamental questions remain unresolved. The metabolic requirements of immune cells change during activation, differentiation, persistence, and antigen recall, and it remains uncertain whether interventions optimized for one functional state will remain beneficial during subsequent transitions. Moreover, simultaneous nutrient deprivation, compensatory pathway activation, intratumoral heterogeneity, and the shared metabolic requirements of malignant and immune cells continue to limit the selectivity and durability of current interventions.

Future progress will require precision strategies guided by the metabolic dependencies of individual tumors and immune-cell populations. Predictive and pharmacodynamic biomarkers will be essential for identifying responsive patients, confirming intratumoral target engagement, and distinguishing causal metabolic vulnerabilities from correlative metabolic signatures. Rational combinations with immune checkpoint blockade or adoptive cell therapy should be designed to remodel the TME without compromising the metabolic fitness of effector lymphocytes. At the same time, cell-specific engineering, localized delivery, and spatially resolved metabolic profiling may help reduce systemic toxicity and address regional heterogeneity within tumors. Ultimately, the most effective metabolic immunotherapies are unlikely to arise from indiscriminate inhibition of individual pathways. Instead, they will depend on a systems-level understanding of metabolic crosstalk and on the selective reprogramming of the tumor–immune metabolic network to support durable anti-tumor immunity.

## Figures and Tables

**Table 1:** Major metabolic pathways in the tumor microenvironment and representative therapeutic targets.

Metabolite/Pathway	Key Metabolic Enzymes	Major Transporters/Receptors	Major Immunological Effects	Representative Therapeutic Targets
Glucose	HK, PKM2, LDHA	GLUT1, GLUT3, MCT1/4	Glucose competition, impaired T cell glycolysis, lactate production	GLUT1, LDHA, MCT1/4
Lactate	LDHA, LDHB	MCT1, MCT4, GPR81, GPR132	CD8^+^ T cell dysfunction, Treg metabolism, TAM M2 polarization	LDHA, MCT1/4, GPR81
Succinate	SDH	SLC25A10, SLC13 family, SUCNR1	HIF-1α stabilization, TAM polarization, angiogenesis	SDH, SUCNR1
Fumarate	FH	(Putative SLC transporters)	KEAP1 succination, NRF2 activation, CD8^+^ T cell suppression	FH, KEAP1–NRF2 axis
2-Hydroxyglutarate (2HG)	Mutant IDH1/2, LDHA	SLC13A3	DNA/histone hypermethylation, impaired T cell activation	IDH1/2
Itaconate	ACOD1 (IRG1)	OXGR1	Macrophage polarization, suppression of CD8^+^ T cell proliferation	ACOD1, OXGR1
Glutamine	GLS	SLC1A5, SLC38A1, SLC38A2	T cell activation, Treg differentiation, anaplerosis	GLS, SLC1A5
Arginine	ARG1, ARG2, NOS	CAT transporters	T cell proliferation, MDSC-mediated immunosuppression	ARG1, ARG2
Serine/Glycine	PHGDH, PSAT1, PSPH, SHMT	ASCT1/2	One-carbon metabolism, CD8^+^ T cell proliferation, Treg function	PHGDH, SHMT
Tryptophan/Kynurenine	IDO1, IDO2, TDO2, AFMID	SLC7A5, SLC7A8, AhR	Treg differentiation, T cell exhaustion, DC dysfunction	IDO1, TDO2, AhR
Methionine	MAT, BHMT, MS	SLC43A2	Epigenetic regulation, T cell survival	SLC43A2, SAM metabolism
Fatty acids	FASN, ACC, CPT1A	CD36, FATPs, FABPs	Lipid accumulation, FAO, Treg/TAM metabolic adaptation	FASN, CPT1A, CD36

**Table 2:** Representative Clinical Trials Evaluating Metabolism-Targeted Interventions Alone or in Combination with Cancer Immunotherapy.

Metabolic Pathway	Target/Agent	Treatment Setting	ClinicalTrials.gov ID	Phase	Current Status and Key Progress
Lactate metabolism	MCT1 inhibitor AZD3965	Advanced solid tumors, diffuse large B-cell lymphoma, and Burkitt lymphoma	NCT01791595	Phase I	Completed. This first-in-human study evaluated the safety, pharmacokinetics, and preliminary activity of MCT1 inhibition. It remains one of the most clinically advanced programs targeting lactate transport, although no confirmatory late-stage study has yet been reported.
Tryptophan–kynurenine pathway	IDO1 inhibitor epacadostat plus pembrolizumab	Unresectable or metastatic melanoma; ECHO-301/KEYNOTE-252	NCT02752074	Phase III	Completed; negative study. A total of 706 participants were enrolled. The addition of epacadostat to pembrolizumab failed to improve clinical outcomes, making this a landmark example of unsuccessful single-axis metabolic checkpoint inhibition.
Tryptophan–kynurenine pathway	IDO1 inhibitor linrodostat mesylate/BMS-986205 plus nivolumab	Recurrent or persistent endometrial cancer or endometrial carcinosarcoma	NCT04106414	Phase II	Active, not recruiting. Twenty-four participants were enrolled, and study completion is estimated for September 2026. No definitive efficacy results have yet been posted.
Tryptophan–kynurenine pathway	Linrodostat mesylate, nivolumab, and perioperative chemotherapy	Muscle-invasive bladder cancer; ENERGIZE	NCT03661320	Phase III	The study record was updated in April 2026. This large perioperative trial represents one of the most advanced late-stage attempts to combine IDO1 inhibition with immune checkpoint blockade and chemotherapy. Public efficacy results remain awaited.
Arginine metabolism	Arginase inhibitor INCB001158/CB-1158 alone or with pembrolizumab	Advanced or metastatic solid tumors	NCT02903914	Phase I/II	Completed; results posted. The study established safety, pharmacokinetic, pharmacodynamic, and recommended-dose information for monotherapy and combination therapy. However, the program has not progressed to a confirmatory late-stage trial.
Glutamine metabolism	Glutaminase inhibitor telaglenastat/CB-839 plus nivolumab	Melanoma, clear-cell renal cell carcinoma, and non-small-cell lung cancer	NCT02771626	Phase I/II	The study evaluated telaglenastat with nivolumab in several tumor cohorts, including patients previously treated with PD-1/PD-L1 inhibitors. The program did not establish a broadly applicable clinical benefit for combined glutaminase and PD-1 blockade.
Glutamine metabolism	Telaglenastat plus pembrolizumab and chemotherapy; KEAPSAKE	First-line KEAP1/NRF2-mutant, nonsquamous NSCLC	NCT04265534	Phase II	Terminated because of lack of clinical benefit. Forty participants were enrolled. Importantly, the study remained negative despite biomarker-based selection for KEAP1/NRF2 alterations.
Glutamine metabolism	Telaglenastat in a molecularly selected basket study; BeGIN	NF1-, KEAP1/NRF2-, or LKB1-aberrant solid tumors	NCT03872427	Phase II	Primary completion occurred in October 2023, with 54 participants enrolled; long-term study completion is estimated for 2027. This trial represents a biomarker-guided attempt to identify tumors with enhanced glutamine dependence.
2-Hydroxyglutarate/IDH1	IDH1 inhibitor ivosidenib plus nivolumab	IDH1-mutant gliomas and advanced solid tumors	NCT04056910	Phase II	Completed. Fifteen patients were enrolled. The trial established the feasibility of combining IDH1 inhibition with PD-1 blockade, but its small sample size limits conclusions regarding clinical efficacy.
Methionine metabolism	Tumor-targeted methioninase delivery using SGN1	Refractory advanced solid tumors	NCT05038150	Phase I/IIa	Recruiting. SGN1 is a modified *Salmonella typhimurium* strain engineered to express L-methioninase and preferentially deplete methionine within tumors. The estimated enrollment is 70, with study completion anticipated in 2028.

Clinical experience indicates that target engagement alone is insufficient to ensure therapeutic efficacy. The failure of epacadostat in the ECHO-301/KEYNOTE-252 trial suggests that inhibition of a single tryptophan-catabolizing enzyme may be inadequate in the absence of biomarker-guided patient selection, confirmation of intratumoral kynurenine suppression, and consideration of compensatory pathways such as TDO2-mediated catabolism [184,185]. Similarly, the limited activity of telaglenastat-containing regimens indicates that genomic alterations associated with glutamine dependence do not invariably predict sustained metabolic vulnerability in vivo [186]. By contrast, IDH inhibitors have achieved clearer clinical success because mutant IDH represents a genetically defined, tumor-selective metabolic dependency with a measurable pharmacodynamic product, D-2HG [187]. These contrasting outcomes emphasize that successful metabolic therapies require a tumor-selective target, reliable biomarkers of pathway dependence and target engagement, and rational combination strategies that preserve immune-cell metabolic fitness.

## Data Availability

No new data were created or analyzed in this study. Data sharing is not applicable to this article.

## Abbreviations

The following abbreviations are used in this manuscript:

TME	Tumor Microenvironment
TAMs	Tumor-Associated Macrophages
MDSCs	Myeloid-Derived Suppressor Cells
Tregs	Regulatory T lymphocytes/cells
TILs	Tumor-Infiltrating Lymphocytes
NK	Natural Killer cells
DCs	Dendritic Cells
CAR-T	Chimeric Antigen Receptor T cells
TCM	Central Memory T cells
TCA	Tricarboxylic Acid (cycle)
OXPHOS	Oxidative Phosphorylation
ROS	Reactive Oxygen Species
2HG	2-Hydroxyglutarate
α-KG	α-Ketoglutarate
GSH	Glutathione
SAM	S-adenosylmethionine
Kyn	Kynurenine
FFAs	Free Fatty Acids
PPP	Pentose Phosphate Pathway
SLC	Solute Carrier (superfamily)
GLUTs	Glucose Transporters
LDHA	Lactate Dehydrogenase A
MCTs	Monocarboxylate Transporters
IDH1/2	Isocitrate Dehydrogenase 1/2
SDH	Succinate Dehydrogenase
FH	Fumarate Hydratase
ACOD1	Aconitate Decarboxylase 1
IDO1	Indoleamine-2, 3-dioxygenase 1
FASN	Fatty Acid Synthase
CPT1A	Carnitine Palmitoyltransferase 1A
FAO	Fatty Acid Oxidation
mTOR	Mammalian Target of Rapamycin
NFAT	Nuclear Factor of Activated T cells
AhR	Aryl Hydrocarbon Receptor
SUCNR1	Succinate Receptor 1
OXGR1	Oxoglutarate Receptor 1
KEAP1	Kelch-like ECH-associated protein 1
NRF2	Nuclear factor erythroid 2-related factor 2
HIF-1α	Hypoxia-inducible factor 1-α
